# Supplementary material for: TORC1 is an essential regulator of nutrient-controlled proliferation and differentiation in Leishmania
Source: EMBO Rep. 2024 Feb 23;25(3):13. doi: 10.1038/s44319-024-00084-y (PMC10933368; doi:10.1038/s44319-024-00084-y)
Supplement: Supplementary file 1 — Appendix [file 44319_2024_84_MOESM1_ESM.pdf]

Appendix (Myburgh et al)

Table of contents

Item	Title	Page
Appendix Figure S1.	Primary and secondary sequences alignments with RPTOR1.	2
Appendix Figure S2.	Flow cytometry analysis of SHERP expression.	3
Appendix Table S1.	Oligonucleotides used in this study.	4
Appendix Table S2.	Plasmids used in this study.	9

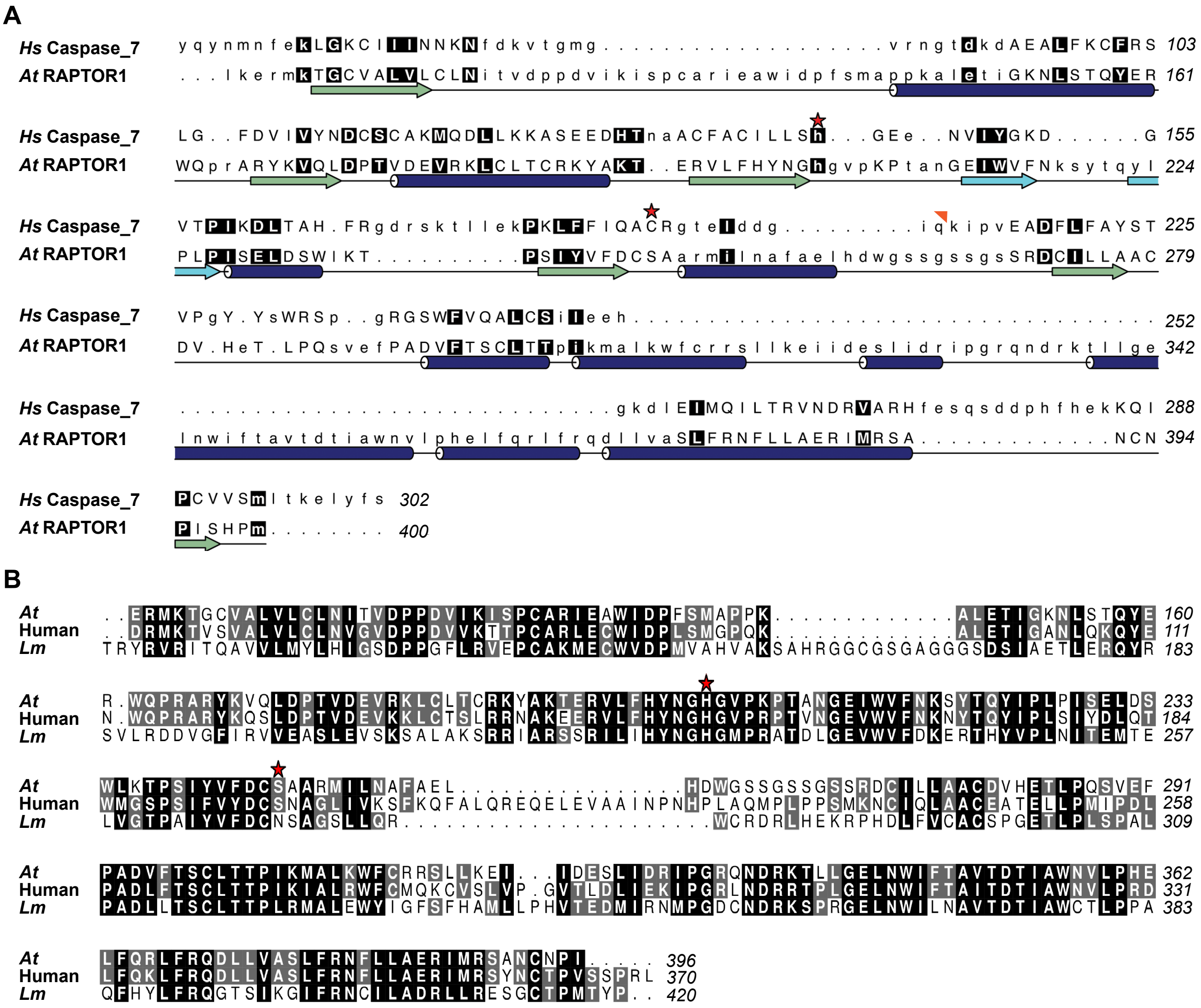

Appendix Figure S1. Primary and secondary sequences alignments with RPTOR1.
A. Sequence alignment based on the overlayed structures of human caspase-7 and the RNC from A. thaliana RAPTOR1, achieved using PDBefold with SSM (Krissinel & Henrick, 2004). The secondary structural elements from At RAPTOR1 were mapped below the alignment, by eye, using ALINE (Bond & Schuttelkopf, 2009).   and secondary structure elements of human caspase-7 and the RNC from A. thaliana RAPTOR1. β-strands are coloured green; the histidine and cysteine residues of the dyad is indicated for caspase-7 by red stars. The position where residues are missing from the caspase 7 structure (cleaved loop, Q196–K212 exclusive) is indicated by an orange triangle. There are no missing residues in this region for AtRAPTOR1. Regions where the structures overlay well are shown in upper case while those whose backbone deviates more or don't align are in lower case. Numbers at the end of each line indicate the position of the last residues in that line in the UniProt sequences.
B. Primary amino acid sequence alignment of A. thaliana RAPTOR1, human RPTOR and L. major RPTOR1. White letters on black background indicate identical residues and white letters on a grey background indicate similar residues; the residues corresponding to the histidine cysteine dyad in caspase-7 shown in (A) are indicated by red stars.

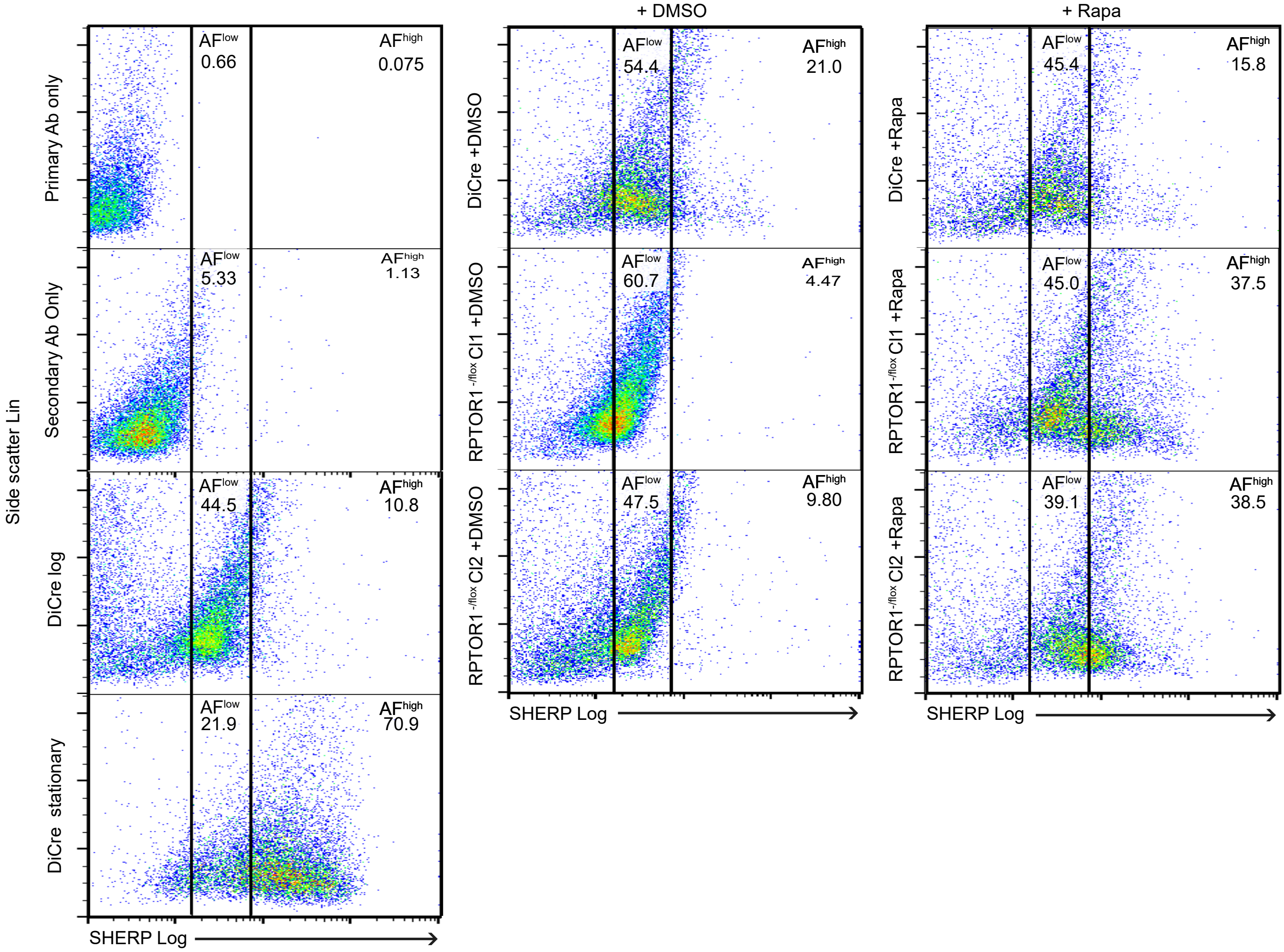

Appendix Figure S2. Flow cytometry analysis of SHERP expression.
SHERP expression was measured by flow cytometry after staining with anti-SHERP and Alexa Fluor 647 (AF647)-conjugated secondary antibodies. Log-stage promastigotes were treated for three days with DMSO or rapamycin (+Rapa), diluted and cultured for three more days with daily DMSO or rapamycin treatment. Representative dot plots of side scatter versus AF647 fluorescence signal (SHERP staining) are shown. Controls (left panel) include DiCre cells stained with primary (1st Ab) or secondary antibody (2nd Ab) only and SHERP-stained DiCre early-log or stationary-phase cells.

Appendix Table S1 - Oligonucleotides used in this study.

Name in figures	Lab Oligo number	F/R	Description	Purpose	Sequence (5'- 3')
P1	OL5040	F	Binds in RPTOR1 CDS. Used to check for WT gene or integration of expression cassette	Diagnostic PCR	GCTTCCAGAACACACCGTA
P2	OL4499	R	Binds RPTOR1 3'UTR. Used to check for cassette integration in RPTOR1 locus	Diagnostic PCR	GTCAAGTGGATTACGGGGAAC
P3	OL14	F	Binds in the 3' region of Hyg resistance gene.Used to check for presence of Hyg resistance cassette.	Diagnostic PCR	CGTCCGAGGGCAAAGGAATA
P4	OL5117	F	Binds in 5' region before RPTOR1 CDS. Used to check for excision of RPTOR1.	Diagnostic PCR	CGTCCTCACTGTTCCTTCTC
P5	OL5118	R	Binds in polyA site after loxP flanked RPTOR1 CDS. Used to check for excision of RPTOR1.	Diagnostic PCR	TCTGCCTCAAACTTGCTTTTC
P6	OL4567	F	Binds in RPTOR1 CDS. Used to amplify *L. major* RPTOR1 coding sequence.	Diagnostic PCR to assess presence of RPTOR1 CDS in ear samples	GCAGCCATGCGCACGAGATTCACTCG
P7	OL4680	R	Binds in RPTOR1 CDS. Used to amplify *L. major* RPTOR1 coding sequence.	Diagnostic PCR to assess presence of RPTOR1 CDS in ear samples	GCAGGCGCAAACGAATAAG
	OL3890	F	Used to amplify an N-terminal fragment of *L. major* RPTOR1 with ***NdeI*** site at 5' end	Generation of RPTOR1 expression plasmid in pET28a for anti-RPTOR1 antibody generation	GC***CATATG***ATTCACTCGTCCACAGCTC
	OL3892	R	Used to amplify an N-terminal fragment of *L. major* RPTOR1 with ***NdeI*** site at 5' end	Generation of RPTOR1 expression plasmid in pET28a for anti-RPTOR1 antibody generation	GACATGATTCGCAACATGCCTTAGAAGCTTGC

OL4392	F	Used to amplify *L. major* RPTOR1 5' flank with PacI site before flank. For generation of Gateway cloning entry vector in pDONR P4-P1R vector	Generation of RPTOR1 knockout construct (pGL2329-Hyg and pGL2330-Sat) through Gateway recombination	GGGGACAACTTTGTATAGAAAAGTTGC***TTAATTAA***CCTAGGAAGAAGCACGGATCTC
OL4323	R	Used to amplify *L. major* RPTOR1 5' flank with PacI site before flank. For generation of Gateway cloning entry vector in pDONR P4-P1R vector	Generation of RPTOR1 knockout construct (pGL2329-Hyg and pGL2330-Sat) through Gateway recombination	GGGGACTGCTTTTTTGTACAAACTTGTGCCCCTGAACAATCCTGTG
OL4324	F	Used to amplify *L. major* RPTOR1 3' flank with PmeI site after flank. For generation of Gateway cloning entry vector in pDONR P2r-P3	Generation of RPTOR1 knockout construct (pGL2329-Hyg and pGL2330-Sat) through Gateway recombination	GGGGACAGCTTTCTTGTACAAAGTGGCTCCCATGCCGGTCTACTG
OL4393	R	Used to amplify *L. major* RPTOR1 3' flank with PmeI site after flank. For generation of Gateway cloning entry vector in pDONR P2r-P3	Generation of RPTOR1 knockout construct (pGL2329-Hyg and pGL2330-Sat) through Gateway recombination	GGGGACAACTTTGTATAATAAAGTTG***GTTTAAAC***GCCAGGCAGCAAGCCATGAG
OL4760	F	Used to amplify RPTOR1 CDS with ***NdeI*** site for cloning into donor vector pGL2015	Generation of floxed RPTOR1 construct (pGL2444-Pur) through Gateway recombination	CA***CATATG***CTGAATCGTCGGCGTAATGCTGATC
OL4761	R	Used to amplify RPTOR1 CDS with ***SpeI*** site for cloning into donor vector pGL2015	Generation of floxed RPTOR1 construct (pGL2444-Pur) through Gateway recombination	TGC***ACTAGT***CAGTTGCAAGAACATGAGTTCG
OL4821	F	Used to amplify untagged RPTOR1 CDS with ***KpnI*** site at 5' end for Gibson assembly with pRib-Neo.	Generation of untagged wildtype RPTOR1 addback plasmid (pGL2765)	ttgagccgtccaccgtagcctcga***GGTACC***ATGCTGAATCGTC
OL4822	R	Used to amplify untagged RPTOR1 CDS with ***XbaI*** site at 3' end for Gibson assembly with pRib-Neo.	Generation of untagged wildtype RPTOR1 addback plasmid (pGL2765)	gccgcggccgcagatcctcaga***TCTAGA***CTACAGTTGCAAGAAC

OL6016	F	Generation of active site mutant of RPTOR1 addback using site-directed mutagenesis.	Generation of untagged and 3x HA-tagged C269A mutant RPTOR1 addback plasmids (pGL2767, pGL2768)	CGTCTTCGACgccAACTCCGCCGGATCCTTG
OL6017	R	Generation of active site mutant of RPTOR1 addback using site-directed mutagenesis.	Generation of untagged and 3x HA-tagged C269A mutant RPTOR1 addback plasmids (pGL2767, pGL2768)	TAGATGGCCGGCGTGCCG
OL6018	F	Generation of HA-tagged RPTOR1 addback using site-directed mutagenesis	Generation of 3x HA-tagged wildtype RPTOR1 addback plasmids (pGL2766)	tcctgactacgcttacccttacgacgtcccggattatgcgTAGTCTAGATCTGAGGATC
OL6019	R	Generation of HA-tagged RPTOR1 addback using site-directed mutagenesis	Generation of 3x HA-tagged wildtype RPTOR1 addback plasmids (pGL2766)	acgtcataggggtatgcgtagtctggcacatcatagggataCAGTTGCAAGAACATGAG
OL7029	F	LmxM.25.0610 _ RPTOR1 Knockout in T7 cell line. Upstream forward primer	Generation of RPTOR1 knockout in *L. mexicana* using CRISPR-CAS9	TAATACGACTCACTATAAAACTGGAAGGGTACTGTGGCCTCGTCGCCCCTGTGTACTTCACTGTGGCCTgtataatgcagacctgctgc
OL7030	R	LmxM.25.0610 _ RPTOR1 Knockout in T7 cell line. Downstream reverse primer.	Generation of RPTOR1 knockout in *L. mexicana* using CRISPR-CAS9	TGGACCAGCCTGGGGGAGTGGGGAAGGGGGccaatttgagagacctgtgc
OL7031	F	LmxM.25.0610 _ RPTOR1 Knockout in T7 cell line_ 5' sgRNA primer	Generation of RPTOR1 knockout in *L. mexicana* using CRISPR-CAS9	gaaattaatacgactcactataggTTCTGGAGAATCTTGTGCAGgttttagagctagaaatagc
OL7032	F	LmxM.25.0610 _ RPTOR1 Knockout in T7 cell line_ 3' sgRNA primer	Generation of RPTOR1 knockout in *L. mexicana* using CRISPR-CAS9	gaaattaatacgactcactataggGCGCCCACACGACGACGGGAgttttagagctagaaatagc
OL9879	F	LmxM.36.6320 (TOR1), N-terminal tagging with mNeonGreen-myc, repair cassette	Generation of TOR1-myc tagged *L. mexicana* line for co-IP	CCCACAAGCGCGCAAGCCATAGGTGGGAGAgtataatgcagacctgctgc
OL9880	R	LmxM.36.6320 (TOR1), N-terminal tagging with mNeonGreen-myc, repair cassette	Generation of TOR1-myc tagged *L. mexicana* line for co-IP	GCTACTGCCTACGCCACCTTTAGACTCCATactacccgatcctgatccag

OL9881	F	LmxM.36.6320 (TOR1), N-terminal tagging with mNeonGreen-myc, sgRNA template	Generation of TOR1-myc tagged *L. mexicana* line for co-IP	gaaattaatacgactcactatag gACGGGAAACAAGCG TTGTAGgttttagagctagaa atagc
OL12508	F	LmxM.29.3580, N-terminal Strep tagging, repair cassette	Generation *L. mexicana* line with strep-tagged proteins for mass spectrometry	TTGACCGCTTTCCCAC AACAGTCGTAGCACgta taatgcagacctgctgc
OL12509	R	LmxM.29.3580, N-terminal Strep tagging, repair cassette	Generation *L. mexicana* line with strep-tagged proteins for mass spectrometry	TGGTTTCCCGTGCTTC TTTGACTTCCCCATaga accggaaccggaacc
OL12510	F	LmxM.29.3580, N-terminal Strep tagging, sgRNA template	Generation *L. mexicana* line with strep-tagged proteins for mass spectrometry	gaaattaatacgactcactatag gCAACACCATCCAGGC GCACAgttttagagctagaaa tagc
OL12511	F	LmxM.25.0610 (RPTOR1), N-terminal Strep tagging, repair cassette	Generation *L. mexicana* line with strep-tagged proteins for mass spectrometry	TCGTCGCCCCTGTGTA CTTCACTGTGGCCTgta taatgcagacctgctgc
OL12512	R	LmxM.25.0610 (RPTOR1), N-terminal Strep tagging, repair cassette	Generation *L. mexicana* line with strep-tagged proteins for mass spectrometry	TCGATCAACATTACGC CGACGATTCAACATag aaccggaaccggaacc
OL12513	F	LmxM.25.0610 (RPTOR1), sgRNA template	Generation *L. mexicana* line with strep-tagged proteins for mass spectrometry	gaaattaatacgactcactatag gTTCTGGAGAATCTTG TGCAGgttttagagctagaaa tagc
OL12514	F	LmxM.36.6320 (TOR1), N-terminal Strep tagging, repair cassette	Generation *L. mexicana* line with strep-tagged proteins for mass spectrometry	CCCACAAGCGCGCAA GCCATAGGTGGGAGA gtataatgcagacctgctgc
OL12515	R	LmxM.36.6320 (TOR1), N-terminal Strep tagging, repair cassette	Generation *L. mexicana* line with strep-tagged proteins for mass spectrometry	GCTACTGCCTACGCC ACCTTTAGACTCCATa gaaccggaaccggaacc
OL12516	F	LmxM.36.6320 (TOR1), sgRNA template	Generation *L. mexicana* line with strep-tagged proteins for mass spectrometry	gaaattaatacgactcactatag gACGGGAAACAAGCG TTGTAGgttttagagctagaa atagc
OL14544	F	LmxM.25.0610 (RPTOR1), C-terminal tagging with mNeonGreen-myc, repair cassette	Generation of RPTOR1-mNeonGreen-myc tagged *L. mexicana* line for localisation	TTTGGCGGCGAGCTC ATGTTCTTGCAACTGg gttctggtagtggttccgg

OL14545	R	LmxM.25.0610 (RPTOR1), C-terminal tagging with mNeonGreen-myc, repair cassette	Generation of RPTOR1-mNeonGreen-myc tagged *L. mexicana* line for localisation	GCGTGTGCGCACCACACAGTCACAGACACGccaatttgagagacctgtgc
OL14546	F	LmxM.25.0610 (RPTOR1), C-terminal tagging with mNeonGreen-myc, sgRNA template	Generation of RPTOR1-mNeonGreen-myc tagged *L. mexicana* line for localisation	gaaattaatacgactcactataggTATCGGACAGTGGACCAGCCgttttagagctagaaatagc
OL11633	F	Used to amplify mouse 5.8S RNA gene	qPCR to determine parasite load in mouse ears	CTCTTAGCGGTGGATCACTC
OL11634	R	Used to amplify mouse 5.8S RNA gene	qPCR to determine parasite load in mouse ears	GTCGATGATCAATGTGTCCTGC
kDNA F2	F	Used to amplify 122 bp product from *Leishmania* kinetoplast DNA minicircle (Acsession nr AF103738.1)	qPCR to determine parasite load in mouse ears	CTCCGGGTAGGGGCGTTC
kDNA R2	R	Used to amplify 122 bp product from *Leishmania* kinetoplast DNA minicircle (Acsession nr AF103738.1)	qPCR to determine parasite load in mouse ears	GCCCTATTTTACACCAACCCC

Appendix Table S2 – Plasmids used in this study.

Plasmid ID	Description	Backbone	Bacterial resistance	Parasite resistance	Reference
pGL2329	*L.major* RPTOR1 knockout contruct with hygromycin resistance gene	pDEST R4-R3	Ampicillin	Hygromycin	N/A
pGL2330	*L.major* RPTOR1 knockout contruct with streptothricin acetyltransferase gene	pDEST R4-R3	Ampicillin	Nourseothricin	N/A
pGL2444	Floxed *L.major* RPTOR1 CDS with C-terminal GFP tag - contains 5' and 3' RPTOR1 flanks to replace gene	pGL2315 (pDONR221)/ pDEST R4-R3	Ampicillin	Puromycin	N/A
pGL2765	Untagged *L.major* RPTOR1 complementation plasmid with neomycin resistance gene	pGL2398 (pRib-Neo)	Ampicillin	Geneticin (G418)	N/A
pGL2766	3x HA-tagged *L.major* RPTOR1 complementation plasmid with neomycin resistance gene	pGL2398 (pRib-Neo)	Ampicillin	Geneticin (G418)	N/A
pGL2767	Untagged *L.major* C269A mutant RPTOR1 complementation plasmid with neomycin resistance gene	pGL2398 (pRib-Neo)	Ampicillin	Geneticin (G418)	N/A
pGL2768	3x HA-tagged *L.major* C269A mutant RPTOR1 complementation plasmid with neomycin resistance gene	pGL2398 (pRib-Neo)	Ampicillin	Geneticin (G418)	N/A
pGL2921	pPLOTv1 twin strep::mNG::twin strep plasmid for amplification of donor DNA for Twin-Strep tagging	pPLOTv1 puro-mCherry-puro	Ampicillin	Puromycin	Backbone obtained from Gluenz lab, Benecke et al, 2017
pGL2666	pPLOTv1 pur::mNG::pur plasmid for amplification of donor DNA for mNeonGreen tagging	pPLOTv1	Ampicillin	Puromycin	Obtained for Gluenz lab, Benecke et al, 2017
pGL2882	pPLOTv1 blast::mNG::blast plasmid for amplification of donor DNA for mNeonGreen and myc tagging	pPLOTv1	Ampicillin	Blasticidin	Obtained for Gluenz lab, Benecke et al, 2017
