# Supplementary figures and images for: TORC1 is an essential regulator of nutrient-controlled proliferation and differentiation in Leishmania
Source: EMBO Rep. 2024 Feb 23;25(3):13. doi: 10.1038/s44319-024-00084-y (PMC10933368; doi:10.1038/s44319-024-00084-y)

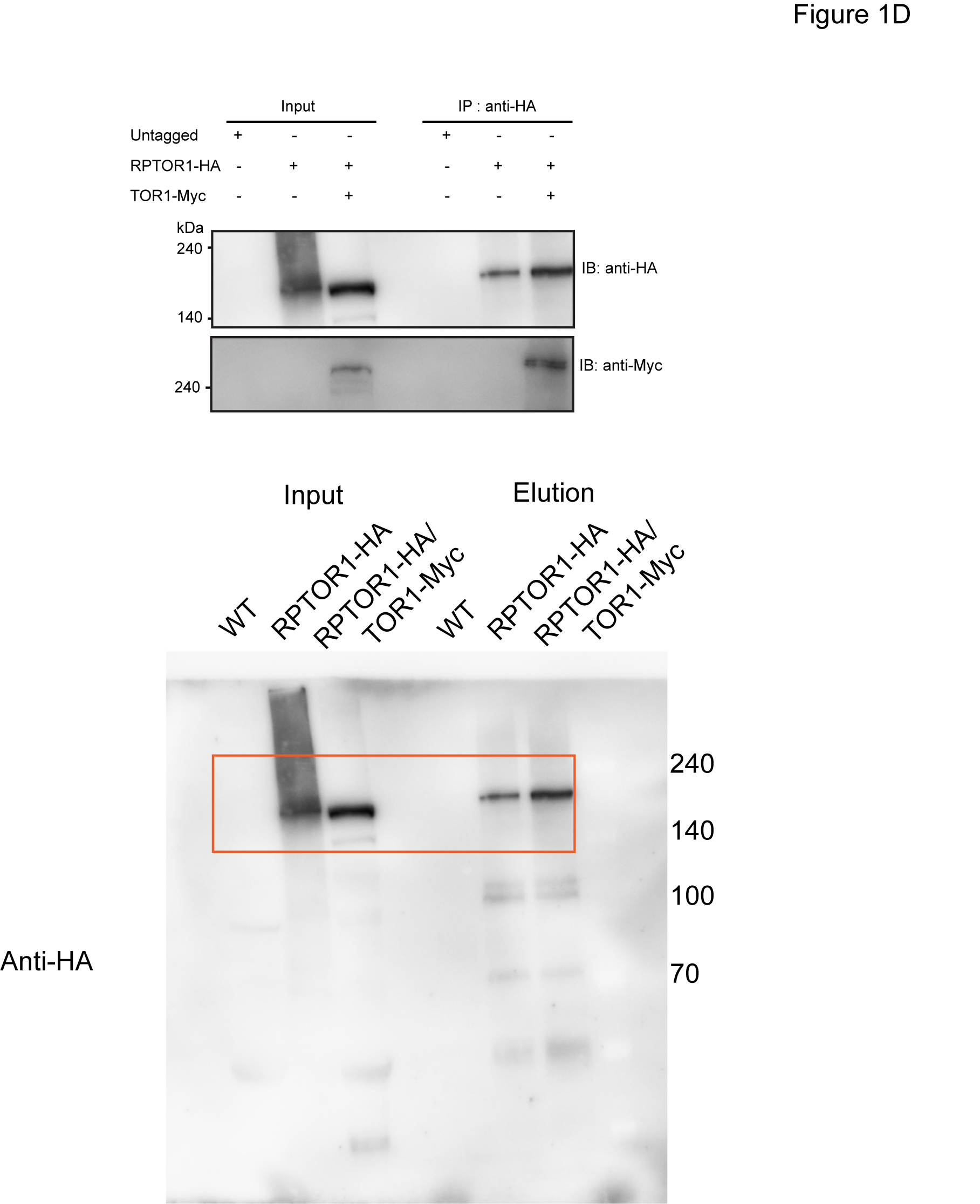

Supplement: Supplementary file 3 — Source Data Fig. 1 [file 44319_2024_84_MOESM3_ESM.zip › 1C/Fig1C_western_HA.tif]

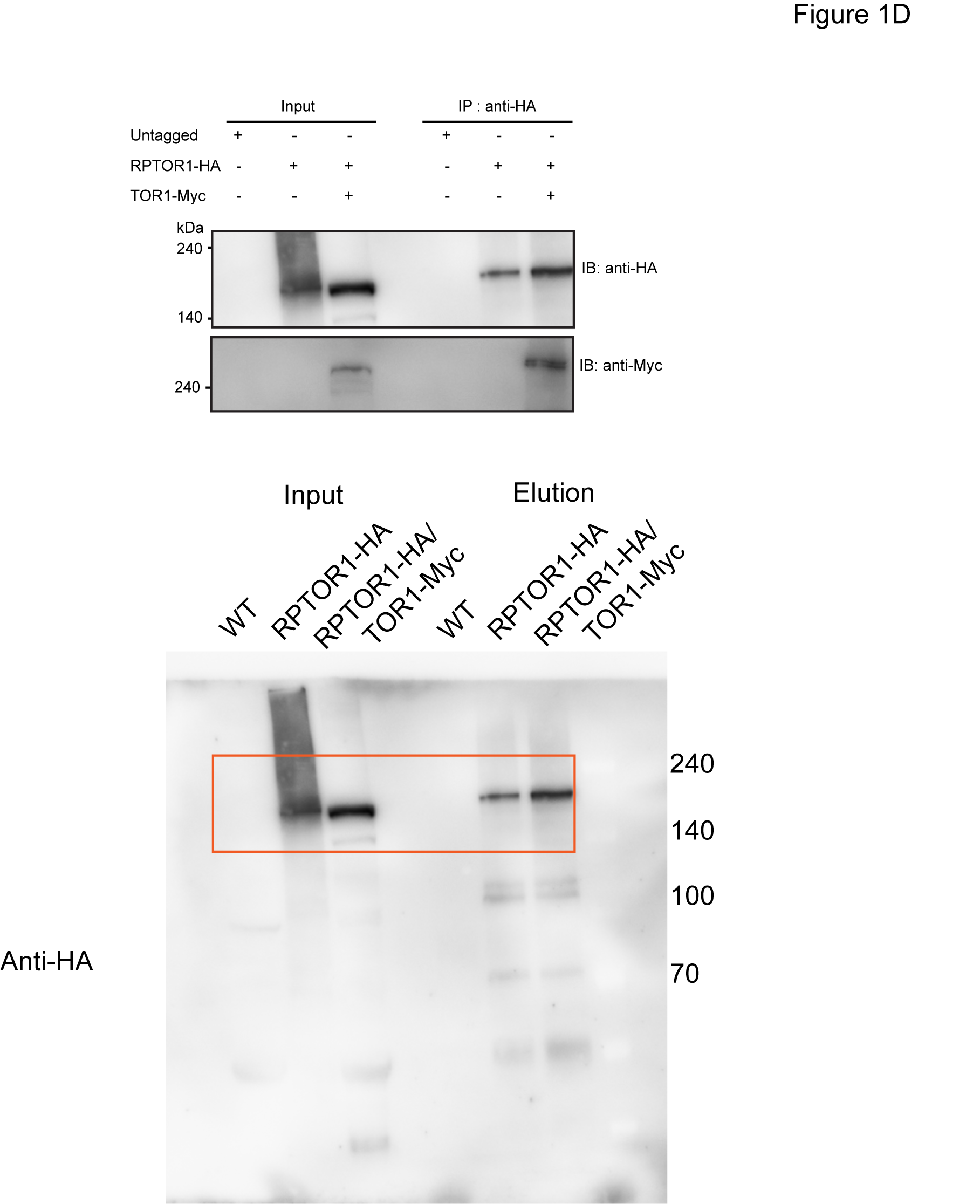

Supplement: Supplementary file 3 — Source Data Fig. 1 [file 44319_2024_84_MOESM3_ESM.zip › 1C/Fig1C_western_myc.tif]

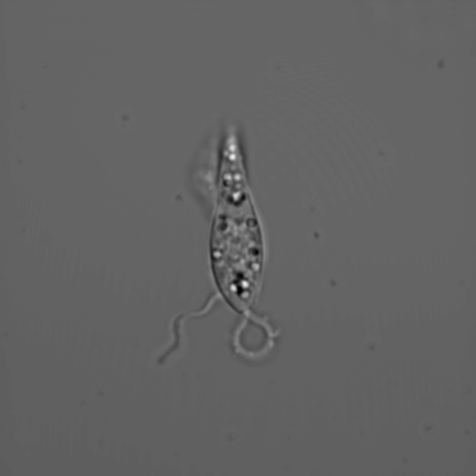

Supplement: Supplementary file 3 — Source Data Fig. 1 [file 44319_2024_84_MOESM3_ESM.zip › 1D/1_DIC_Figure1D.tif]

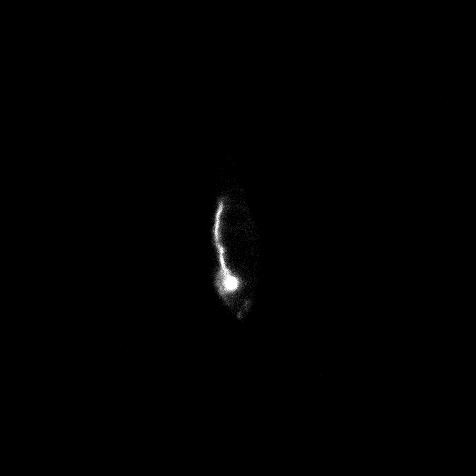

Supplement: Supplementary file 3 — Source Data Fig. 1 [file 44319_2024_84_MOESM3_ESM.zip › 1D/1_FM-4-64_Figure1D.tif]

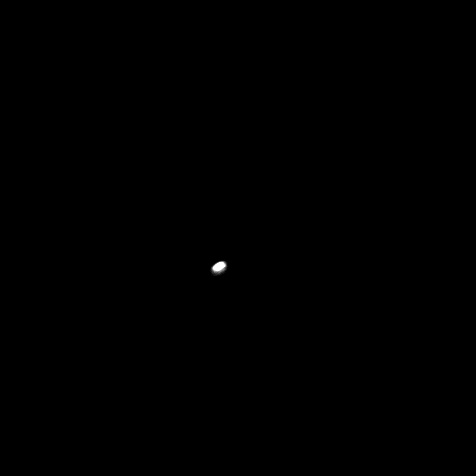

Supplement: Supplementary file 3 — Source Data Fig. 1 [file 44319_2024_84_MOESM3_ESM.zip › 1D/1_Hoechst_Figure1D.tif]

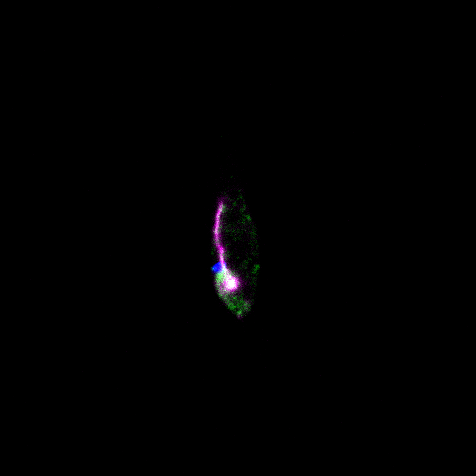

Supplement: Supplementary file 3 — Source Data Fig. 1 [file 44319_2024_84_MOESM3_ESM.zip › 1D/1_merge_Figure1D.tif]

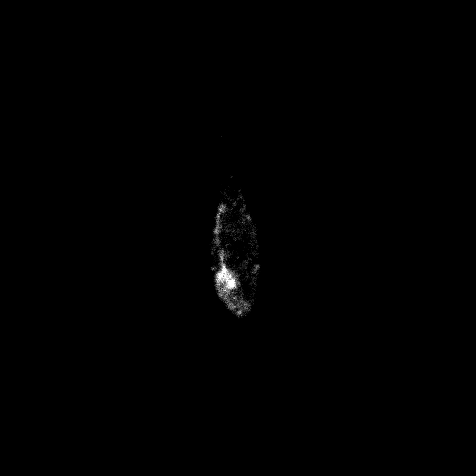

Supplement: Supplementary file 3 — Source Data Fig. 1 [file 44319_2024_84_MOESM3_ESM.zip › 1D/1_mNeonGreen_Figure1D.tif]

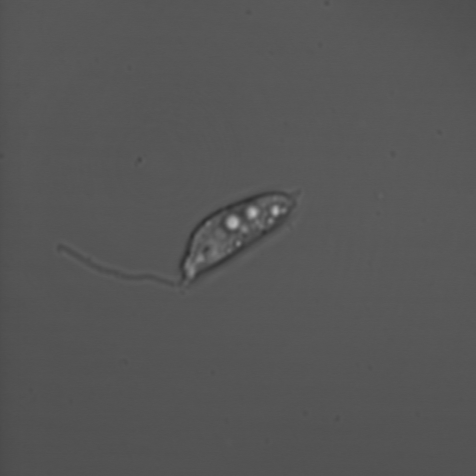

Supplement: Supplementary file 3 — Source Data Fig. 1 [file 44319_2024_84_MOESM3_ESM.zip › 1D/2_DIC_Figure1D.tif]

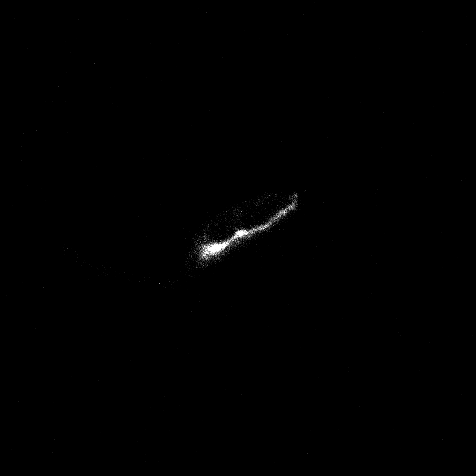

Supplement: Supplementary file 3 — Source Data Fig. 1 [file 44319_2024_84_MOESM3_ESM.zip › 1D/2_FM-4-64_Figure1D.tif]

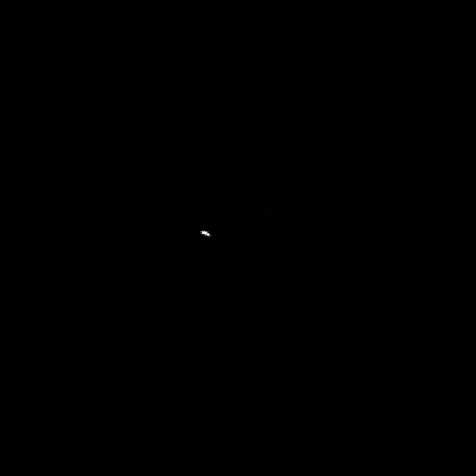

Supplement: Supplementary file 3 — Source Data Fig. 1 [file 44319_2024_84_MOESM3_ESM.zip › 1D/2_Hoechst_Figure1D.tif]

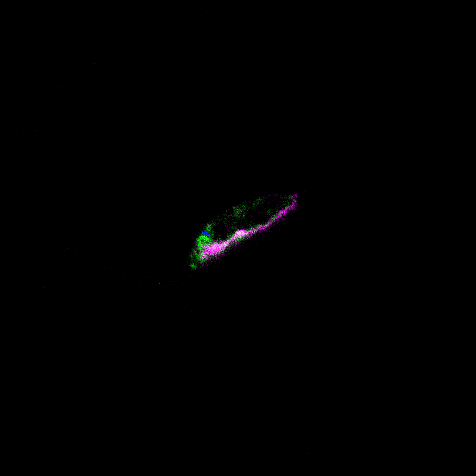

Supplement: Supplementary file 3 — Source Data Fig. 1 [file 44319_2024_84_MOESM3_ESM.zip › 1D/2_Merge_Figure1D.tif]

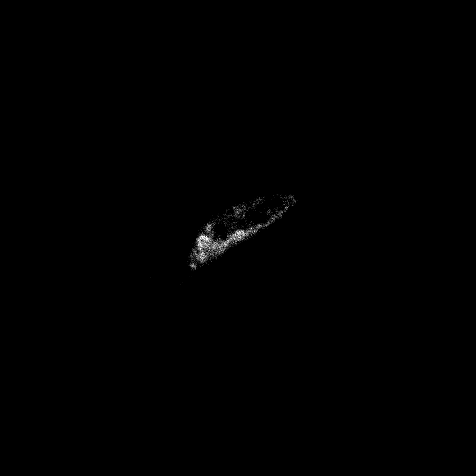

Supplement: Supplementary file 3 — Source Data Fig. 1 [file 44319_2024_84_MOESM3_ESM.zip › 1D/2_mNeonGreen_Figure1D.tif]

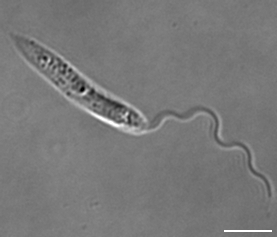

Supplement: Supplementary file 3 — Source Data Fig. 1 [file 44319_2024_84_MOESM3_ESM.zip › 1D/3_DIC_Figure1D.tif]

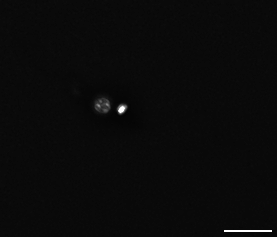

Supplement: Supplementary file 3 — Source Data Fig. 1 [file 44319_2024_84_MOESM3_ESM.zip › 1D/3_Hoechst_Figure1D.tif]

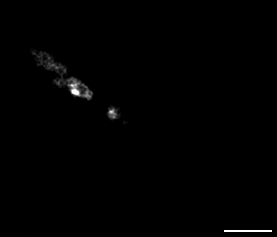

Supplement: Supplementary file 3 — Source Data Fig. 1 [file 44319_2024_84_MOESM3_ESM.zip › 1D/3_Lysotracker_Figure1D.tif]

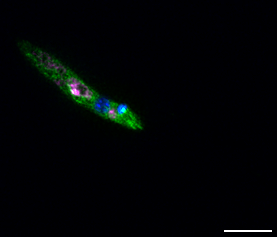

Supplement: Supplementary file 3 — Source Data Fig. 1 [file 44319_2024_84_MOESM3_ESM.zip › 1D/3_Merge_Figure1D.tif]

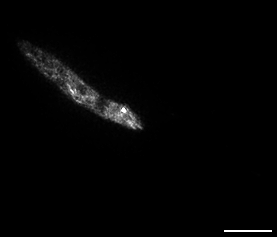

Supplement: Supplementary file 3 — Source Data Fig. 1 [file 44319_2024_84_MOESM3_ESM.zip › 1D/3_mNeonGreen_Figure1D.tif]

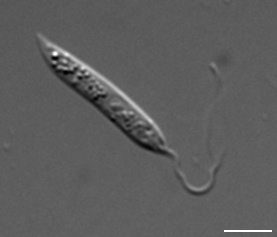

Supplement: Supplementary file 3 — Source Data Fig. 1 [file 44319_2024_84_MOESM3_ESM.zip › 1D/4_DIC_Figure1D.tif]

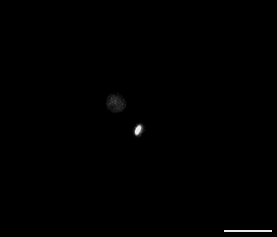

Supplement: Supplementary file 3 — Source Data Fig. 1 [file 44319_2024_84_MOESM3_ESM.zip › 1D/4_Hoechst_Figure1D.tif]

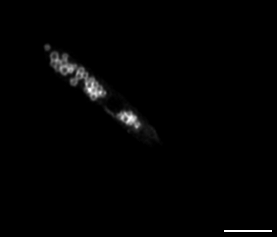

Supplement: Supplementary file 3 — Source Data Fig. 1 [file 44319_2024_84_MOESM3_ESM.zip › 1D/4_Lysotracker_Figure1D.tif]

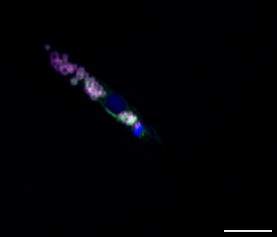

Supplement: Supplementary file 3 — Source Data Fig. 1 [file 44319_2024_84_MOESM3_ESM.zip › 1D/4_merge_Figure1D.tif]

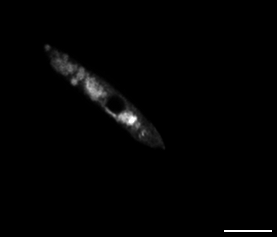

Supplement: Supplementary file 3 — Source Data Fig. 1 [file 44319_2024_84_MOESM3_ESM.zip › 1D/4_mNeonGreen_Figure1D.tif]

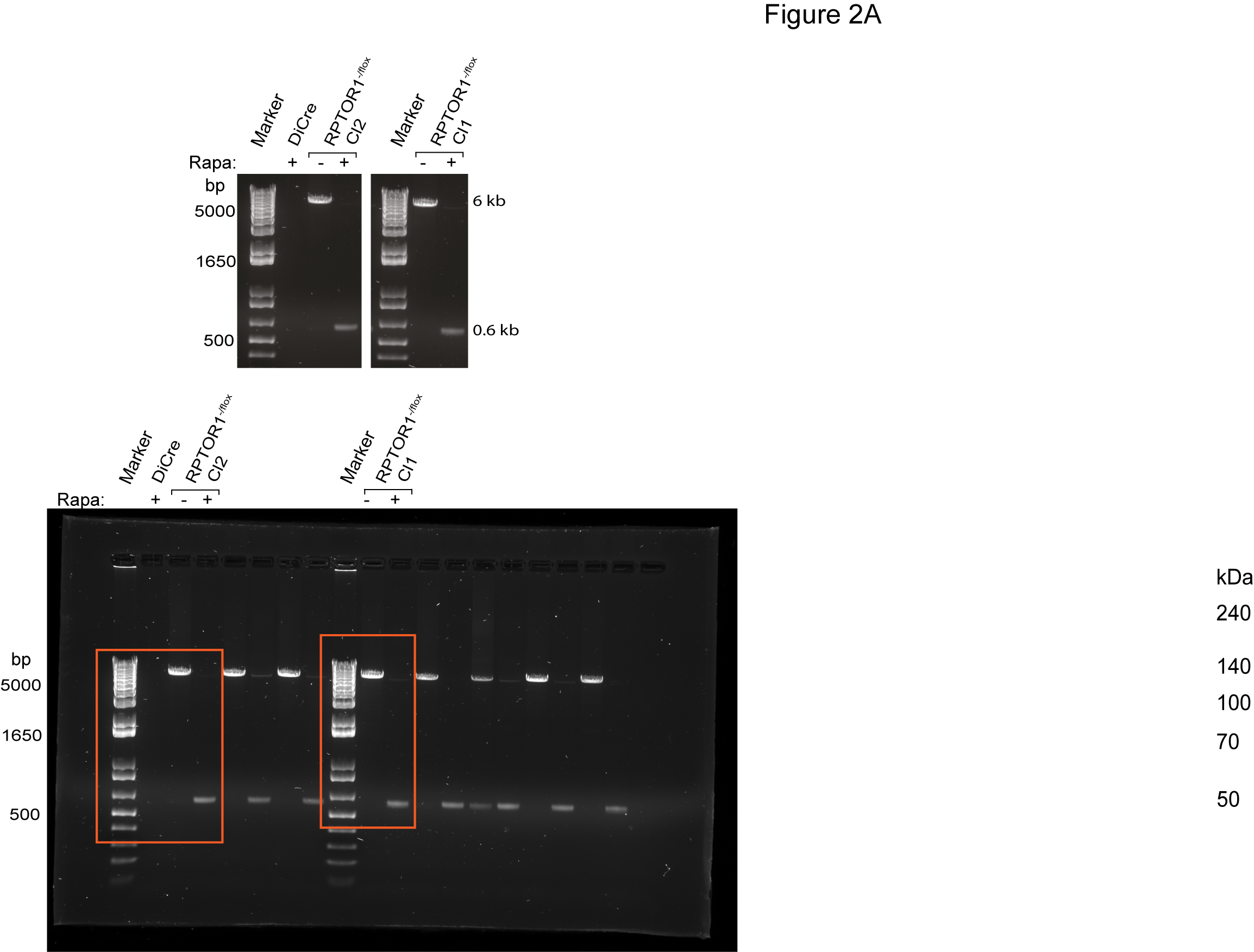

Supplement: Supplementary file 4 — Source Data Fig. 2 [file 44319_2024_84_MOESM4_ESM.zip › 2A/excision_PCR_gel_Figure2A.tif]

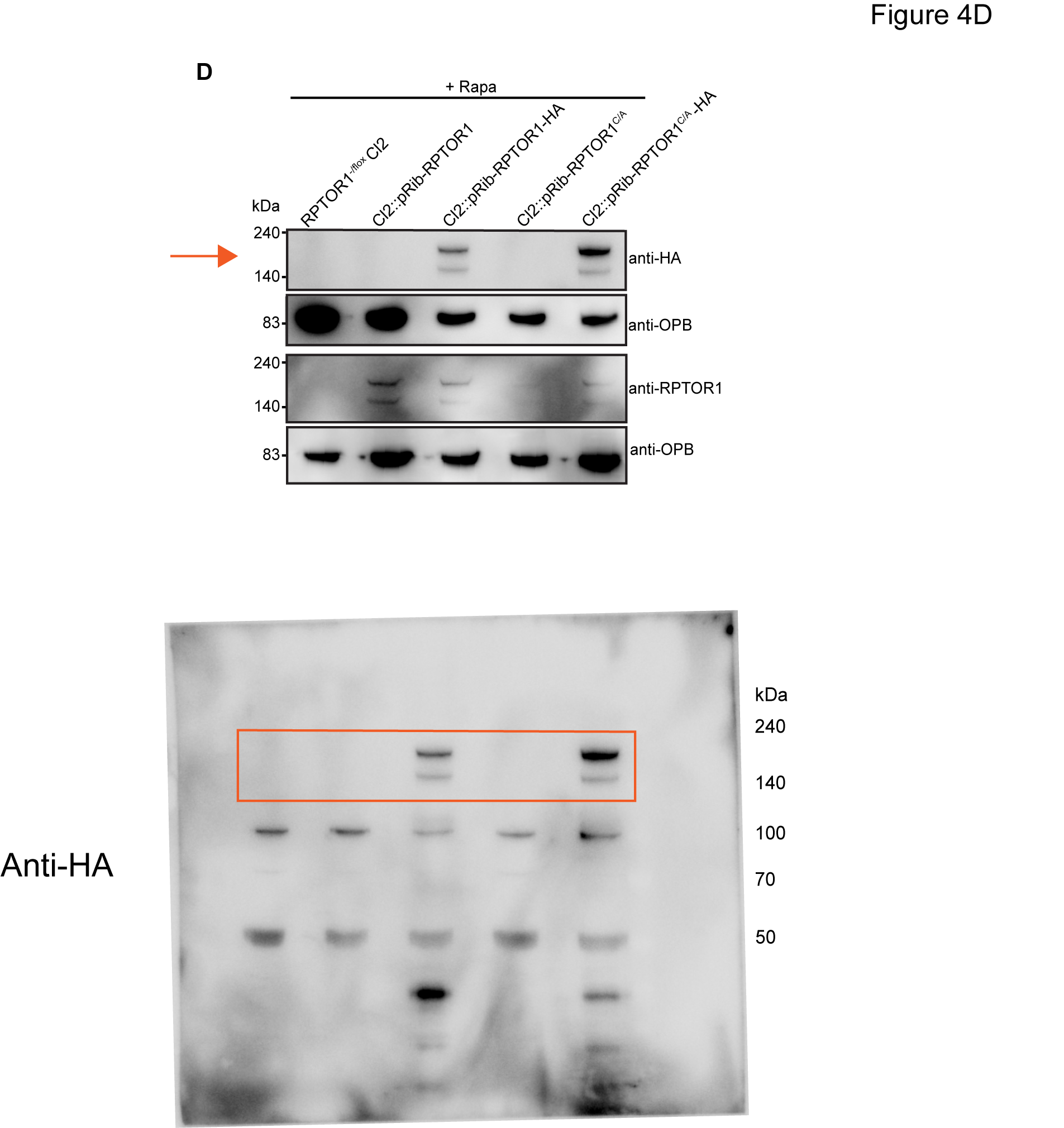

Supplement: Supplementary file 6 — Source Data Fig. 4 [file 44319_2024_84_MOESM6_ESM.zip › 4D/Fig4D_1_western_HA.tif]

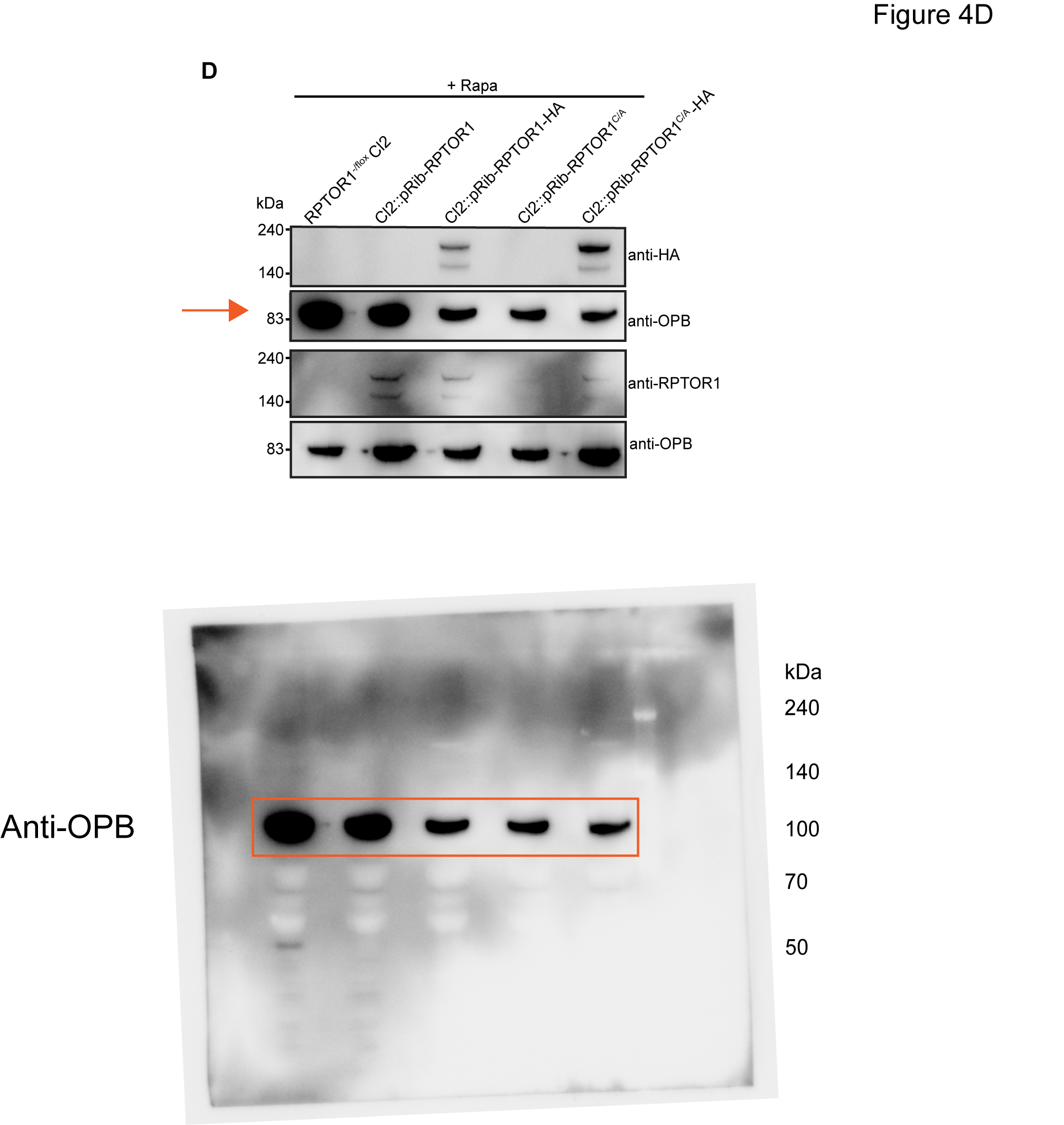

Supplement: Supplementary file 6 — Source Data Fig. 4 [file 44319_2024_84_MOESM6_ESM.zip › 4D/Fig4D_2_western_OPBforHA.tif]

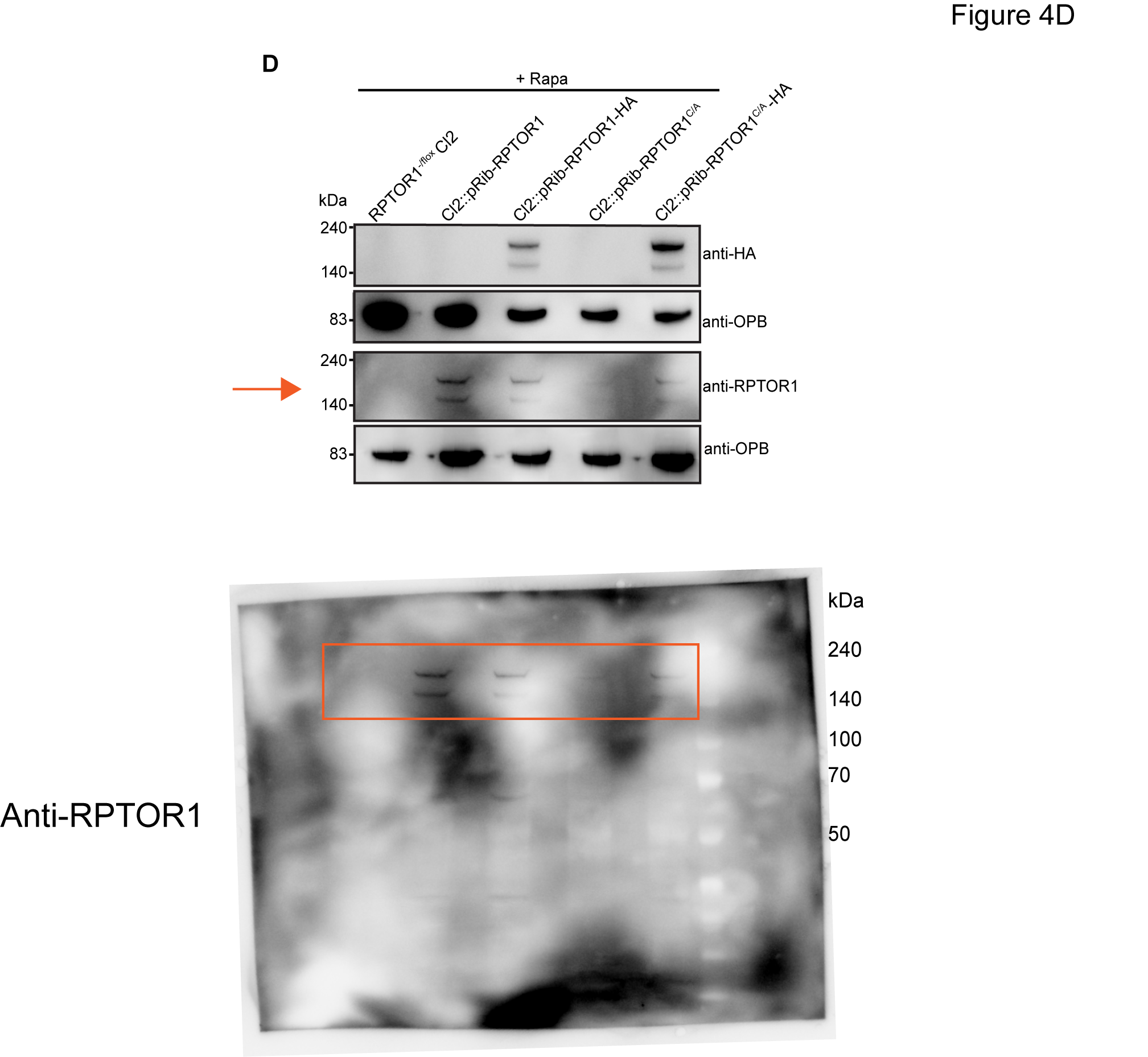

Supplement: Supplementary file 6 — Source Data Fig. 4 [file 44319_2024_84_MOESM6_ESM.zip › 4D/Fig4D_3_western_RPTOR1.tif]

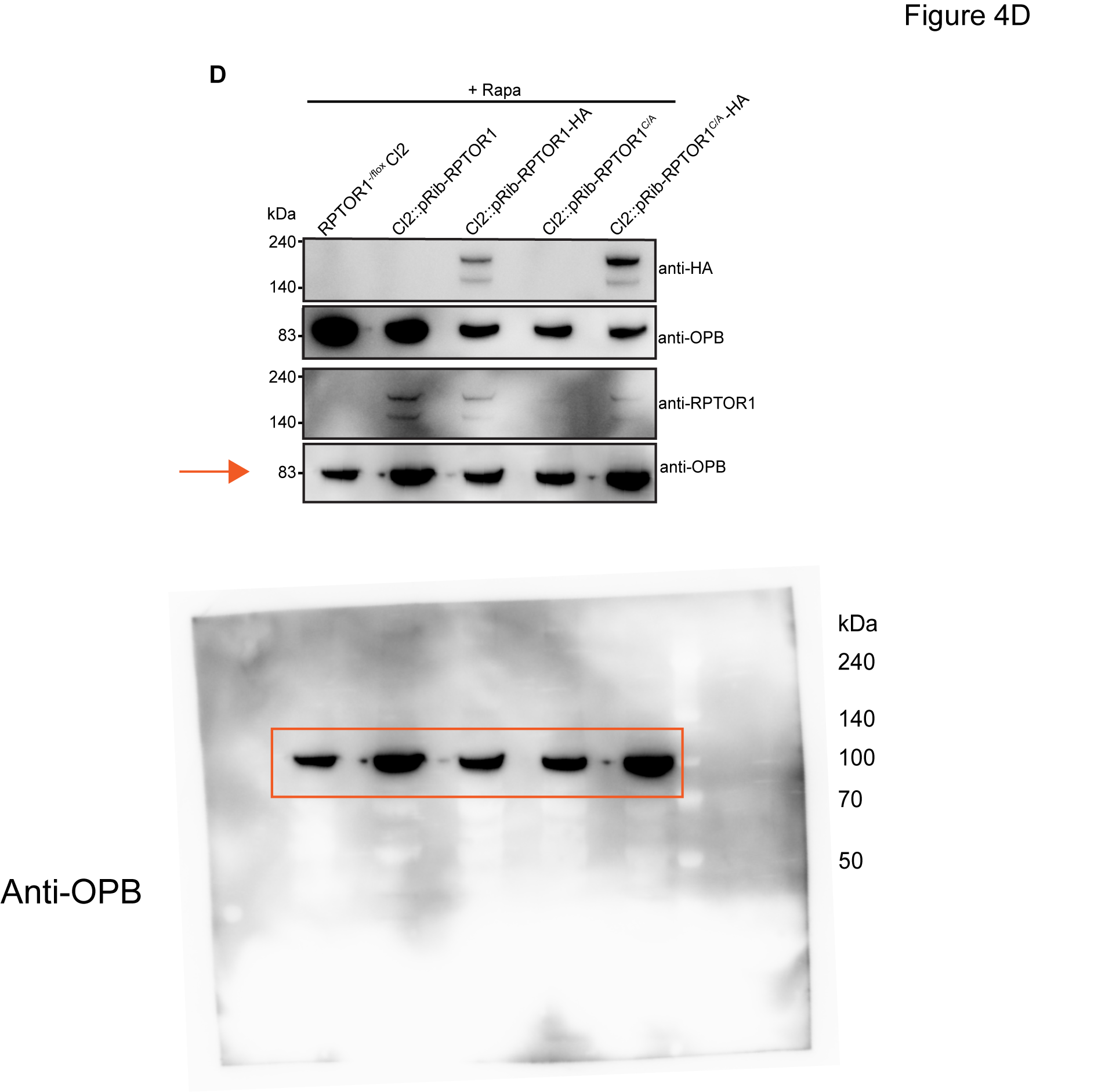

Supplement: Supplementary file 6 — Source Data Fig. 4 [file 44319_2024_84_MOESM6_ESM.zip › 4D/Fig4D_4_western_OPBforRPTOR1.tif]

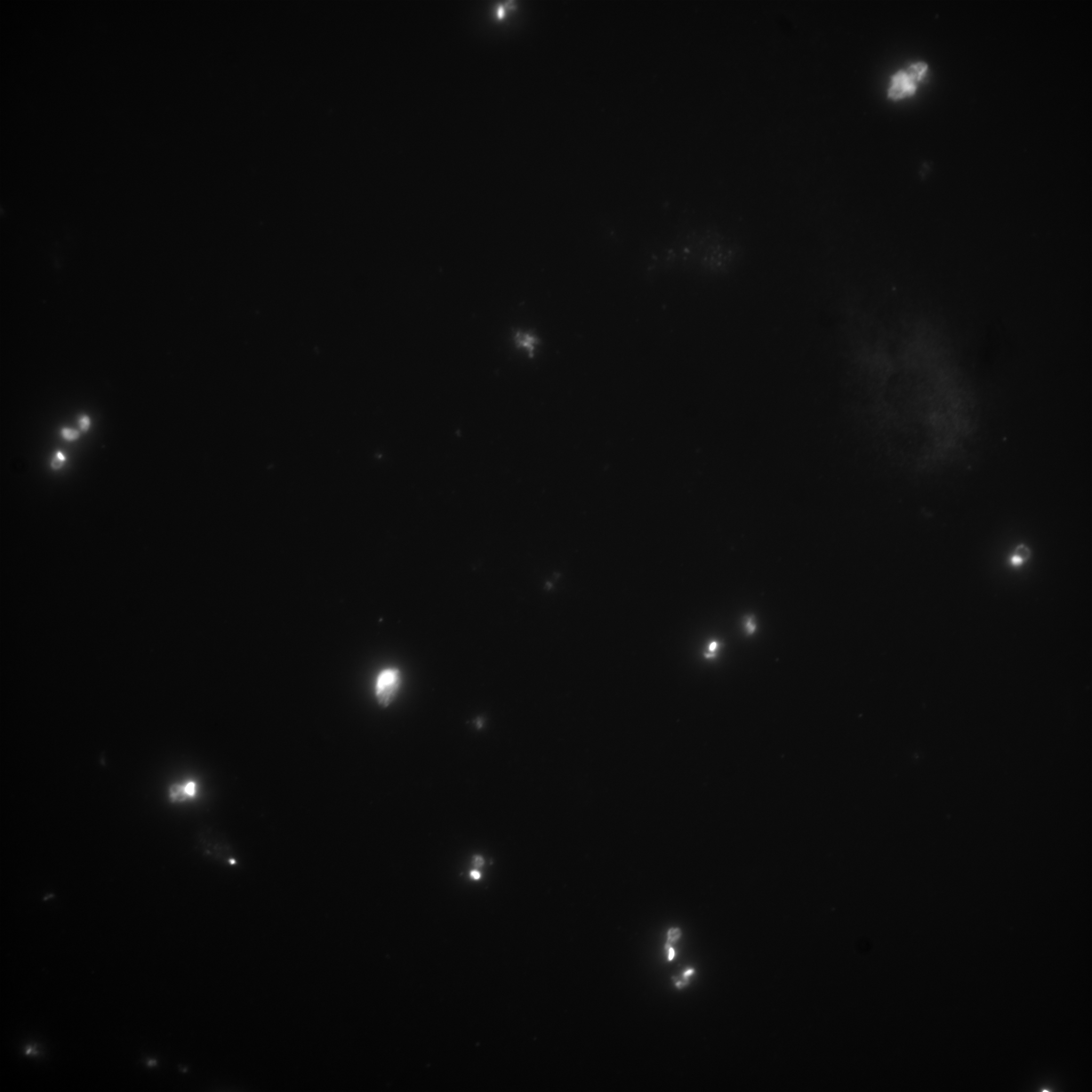

Supplement: Supplementary file 8 — Source Data Fig. 6 [file 44319_2024_84_MOESM8_ESM.zip › 6B/DiCre_DAPI_Fig6B.tif]

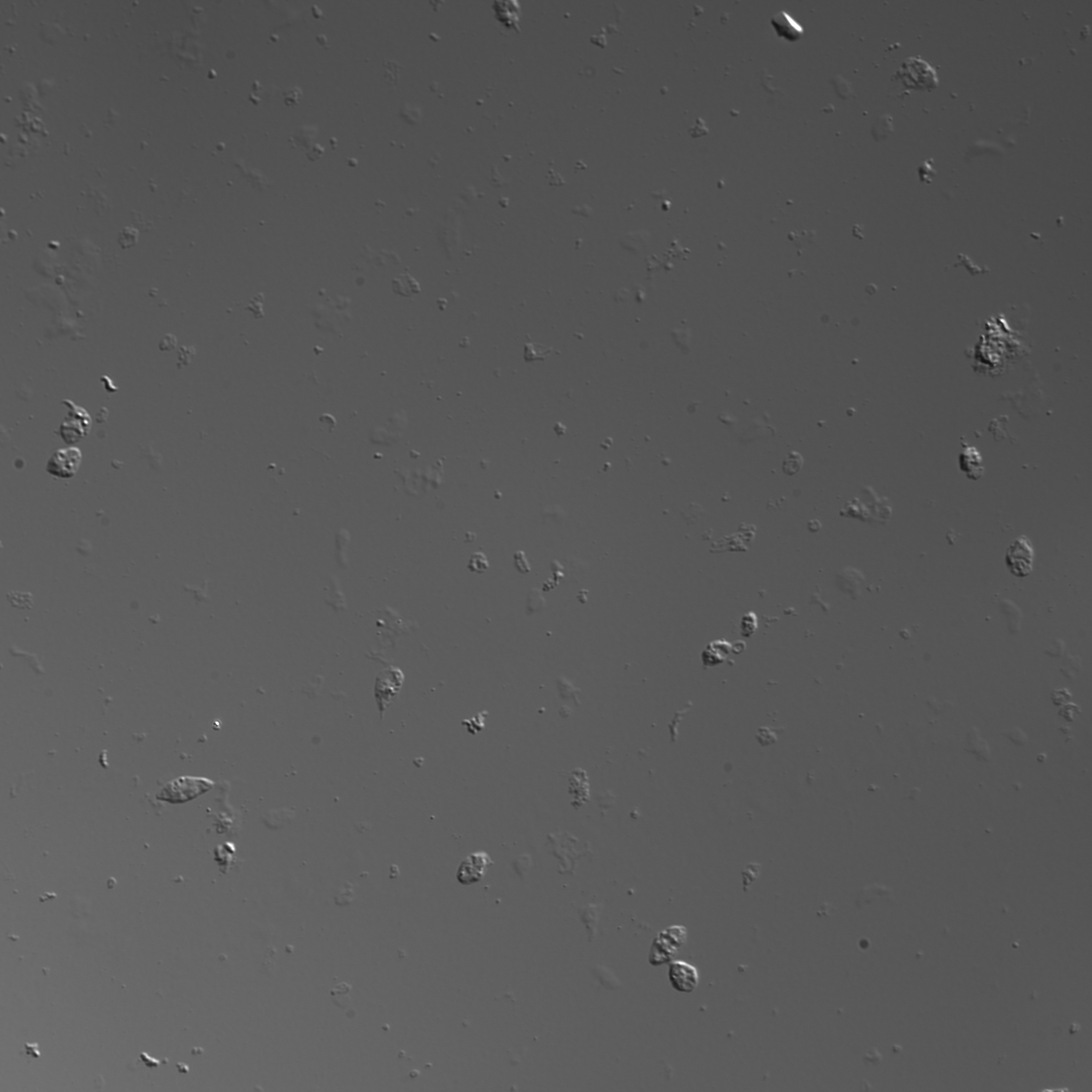

Supplement: Supplementary file 8 — Source Data Fig. 6 [file 44319_2024_84_MOESM8_ESM.zip › 6B/DiCre_DIC_Fig6B.tif]

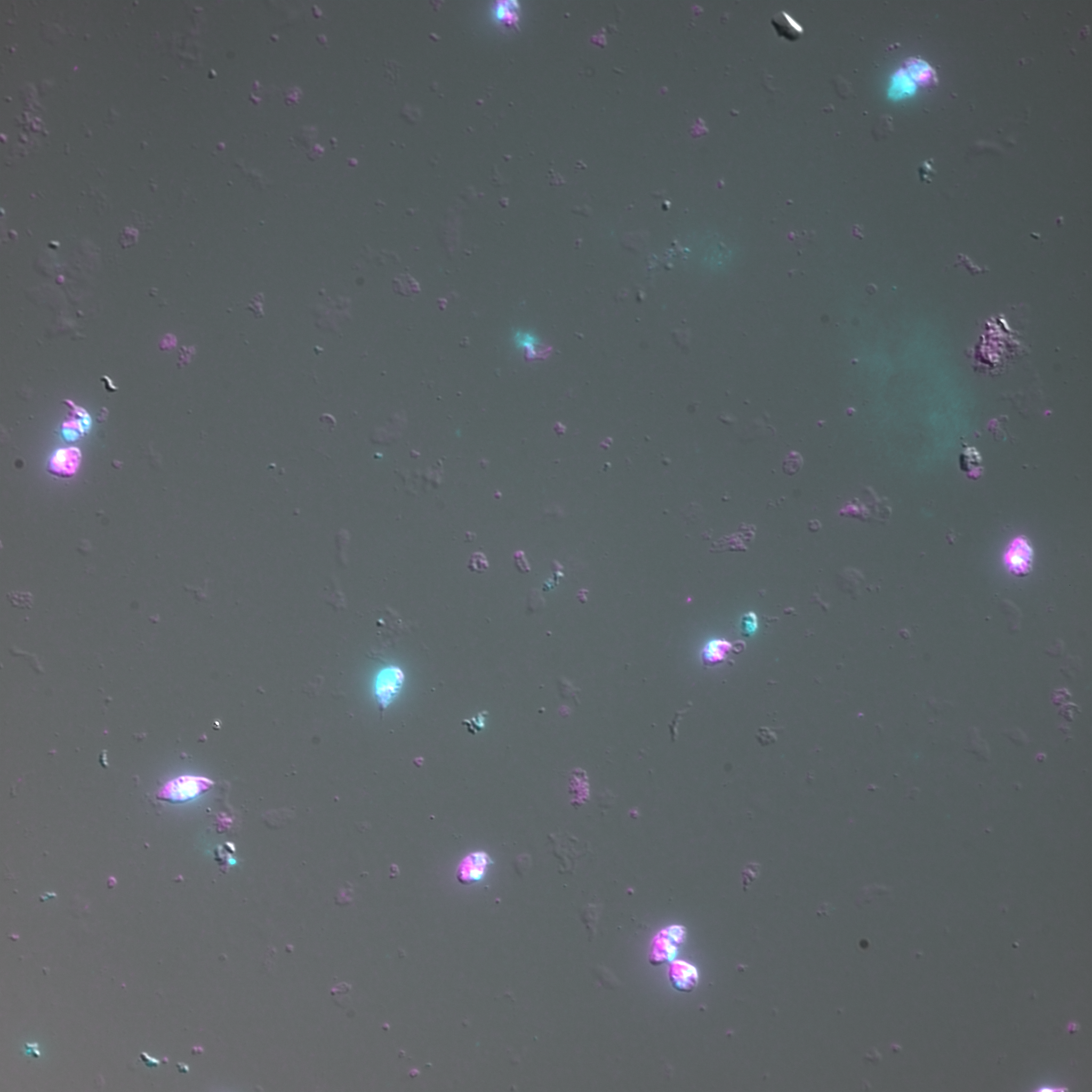

Supplement: Supplementary file 8 — Source Data Fig. 6 [file 44319_2024_84_MOESM8_ESM.zip › 6B/DiCre_Merge_Fig6B.tif]

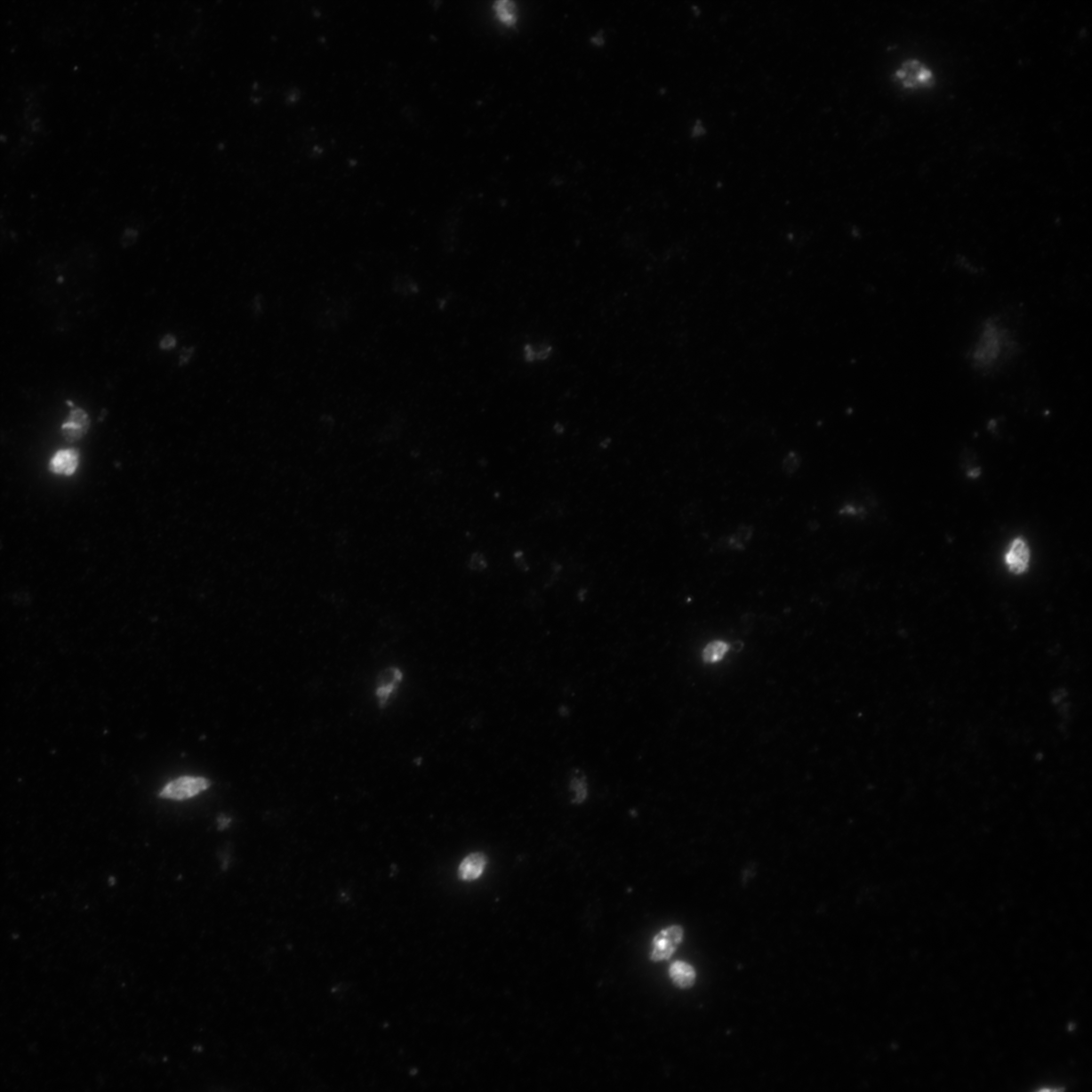

Supplement: Supplementary file 8 — Source Data Fig. 6 [file 44319_2024_84_MOESM8_ESM.zip › 6B/DiCre_OPB_Fig6B.tif]

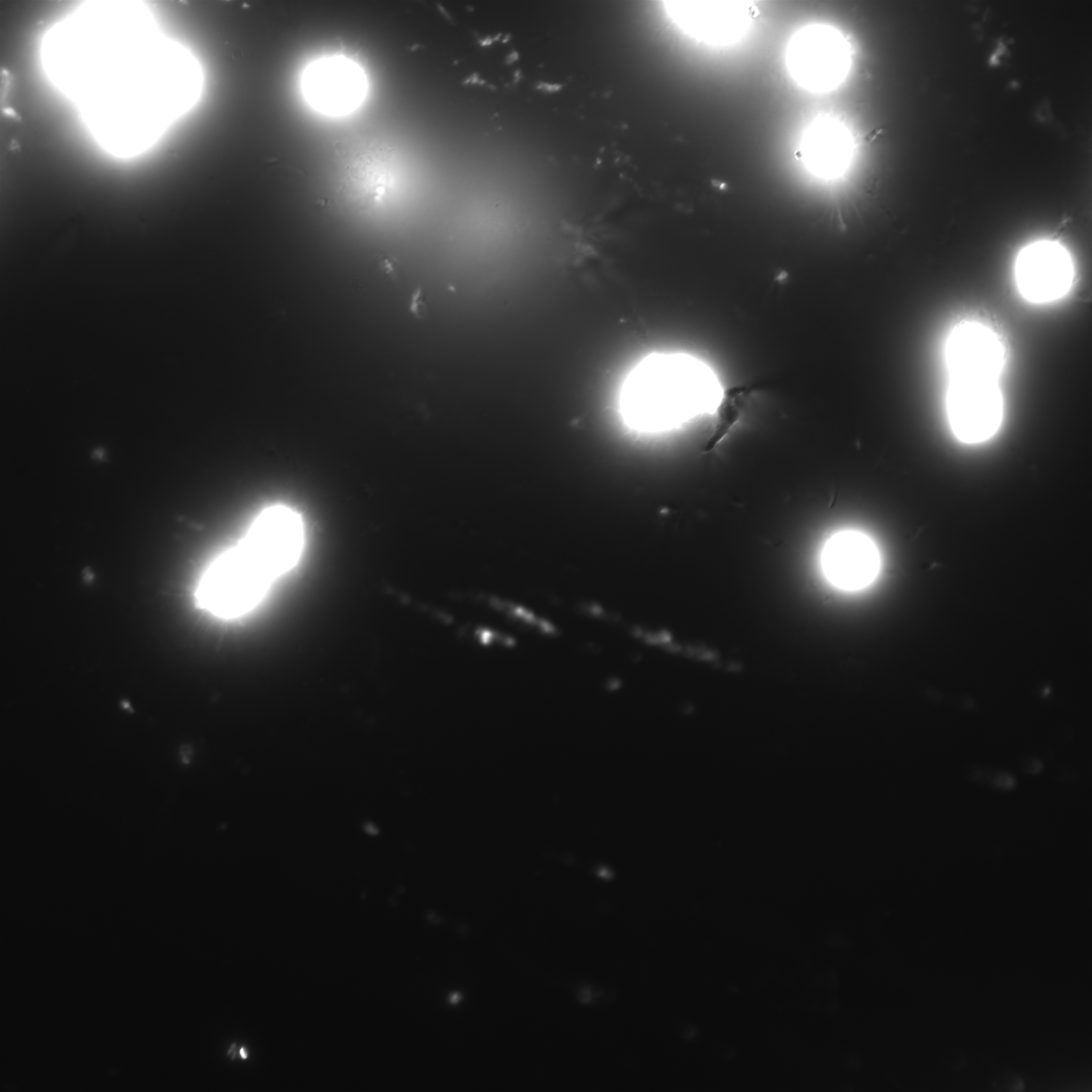

Supplement: Supplementary file 8 — Source Data Fig. 6 [file 44319_2024_84_MOESM8_ESM.zip › 6B/RPTOR1_Cl2_1_DAPI_Fig6B.tif]

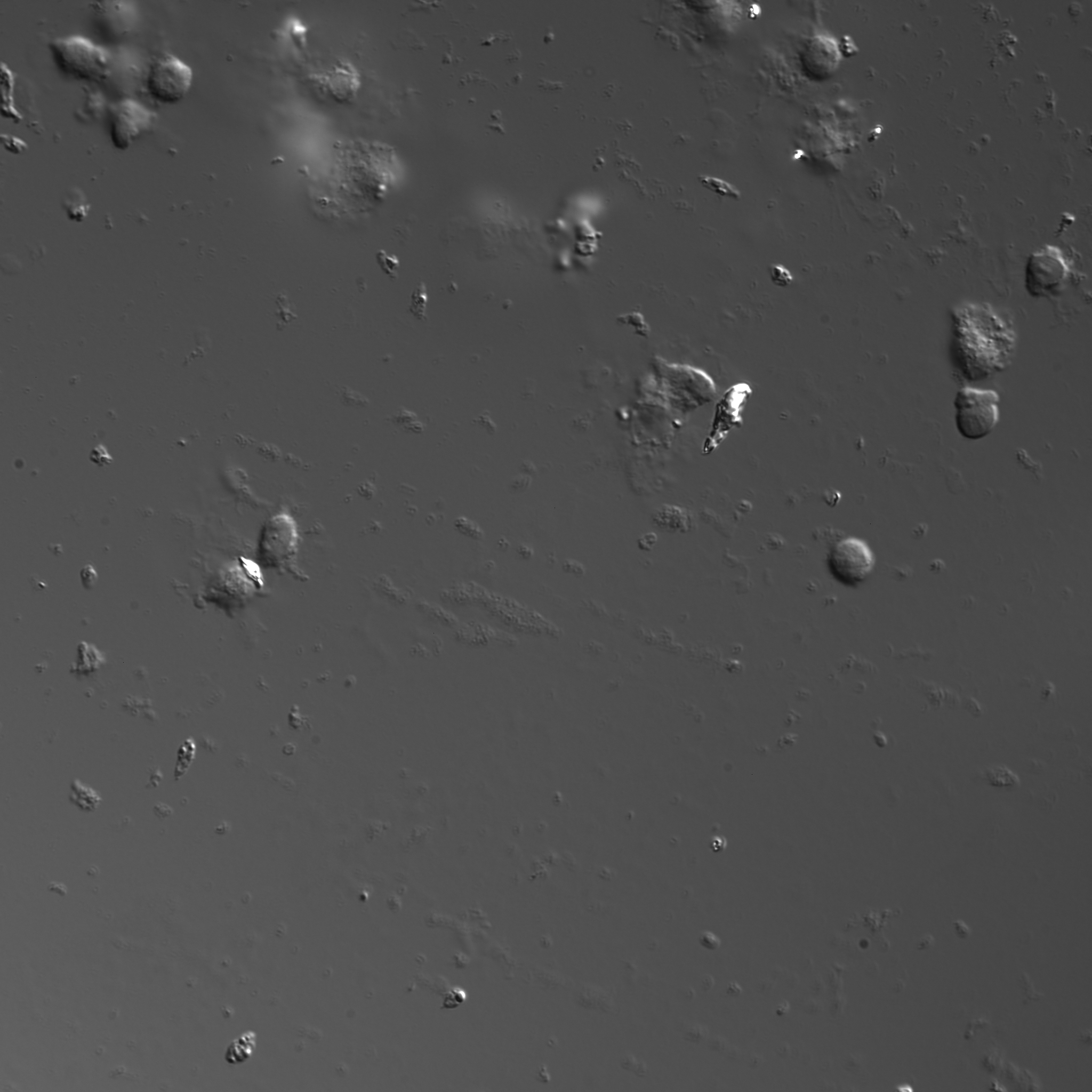

Supplement: Supplementary file 8 — Source Data Fig. 6 [file 44319_2024_84_MOESM8_ESM.zip › 6B/RPTOR1_Cl2_1_DIC_Fig6B.tif]

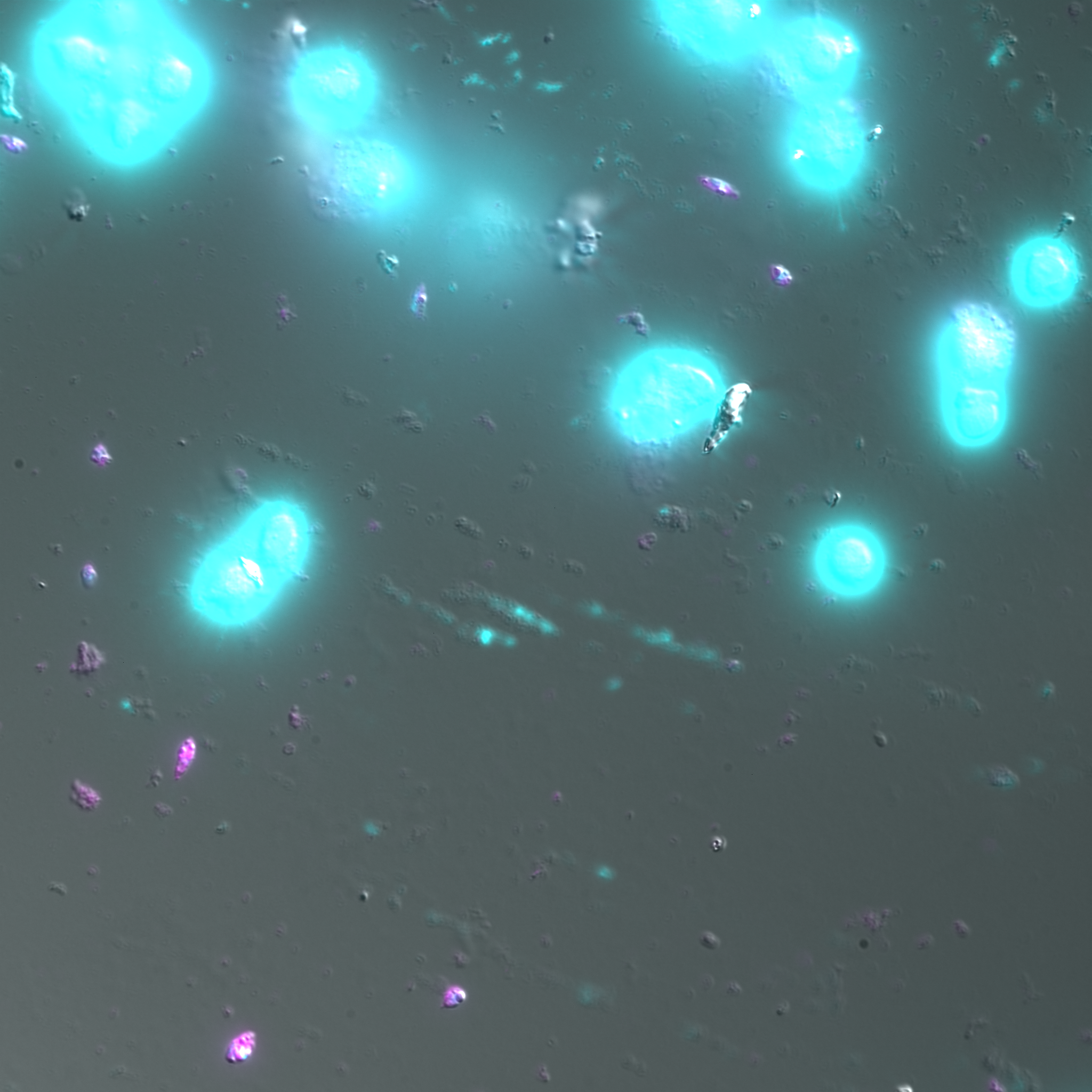

Supplement: Supplementary file 8 — Source Data Fig. 6 [file 44319_2024_84_MOESM8_ESM.zip › 6B/RPTOR1_Cl2_1_Merge_Fig6B.tif]

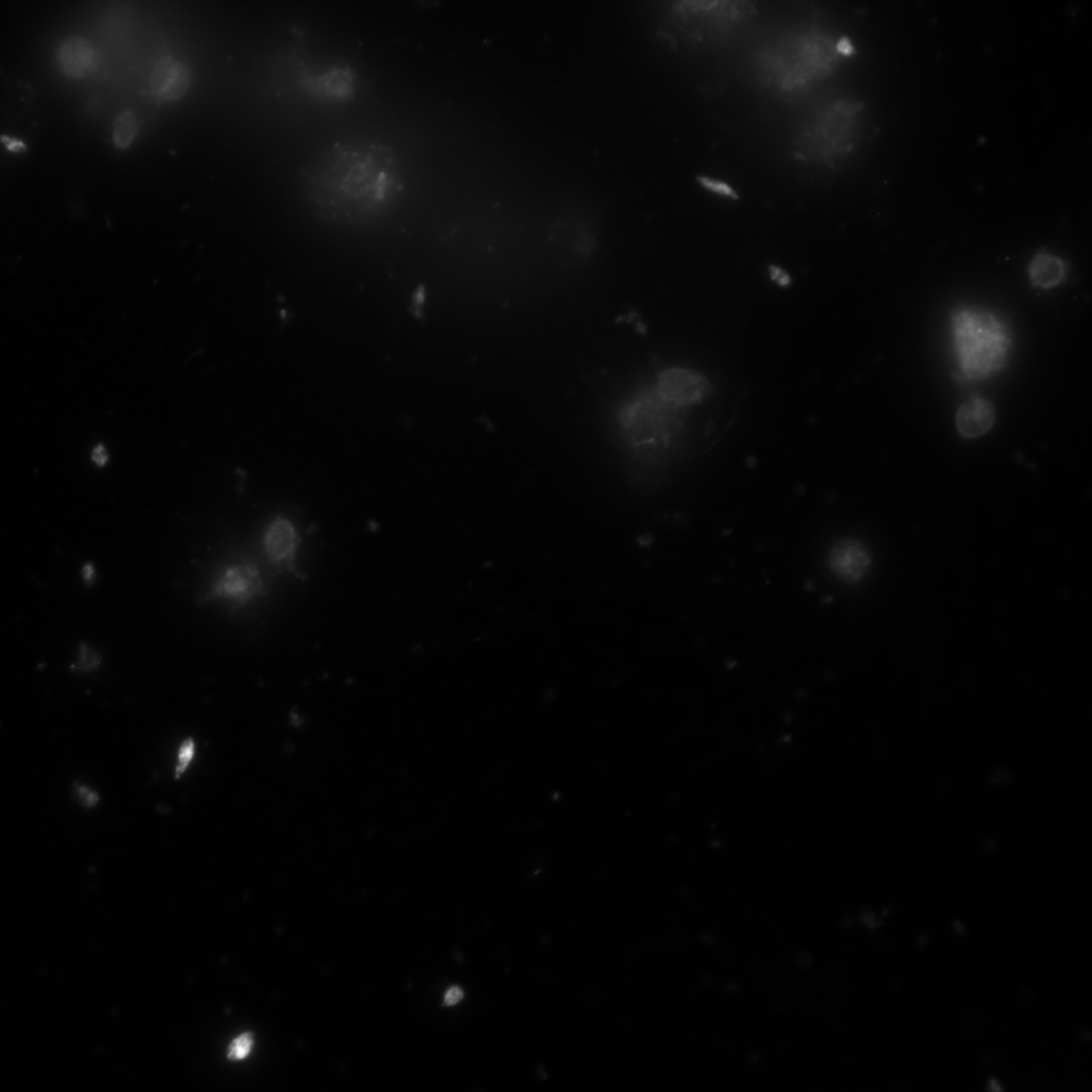

Supplement: Supplementary file 8 — Source Data Fig. 6 [file 44319_2024_84_MOESM8_ESM.zip › 6B/RPTOR1_Cl2_1_OPB_Fig6B.tif]

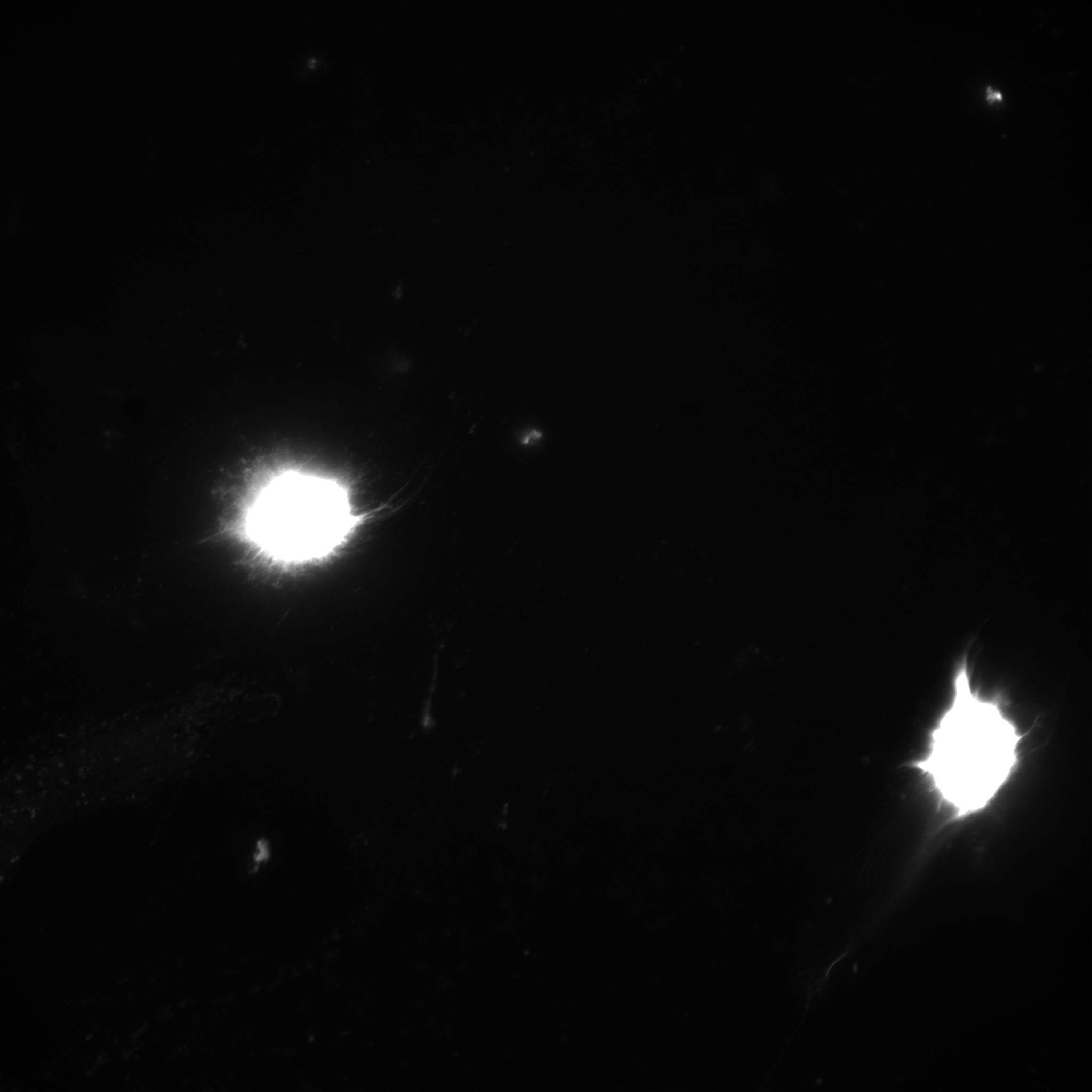

Supplement: Supplementary file 8 — Source Data Fig. 6 [file 44319_2024_84_MOESM8_ESM.zip › 6B/RPTOR1_Cl2_2_DAPI_Fig6B.tif]

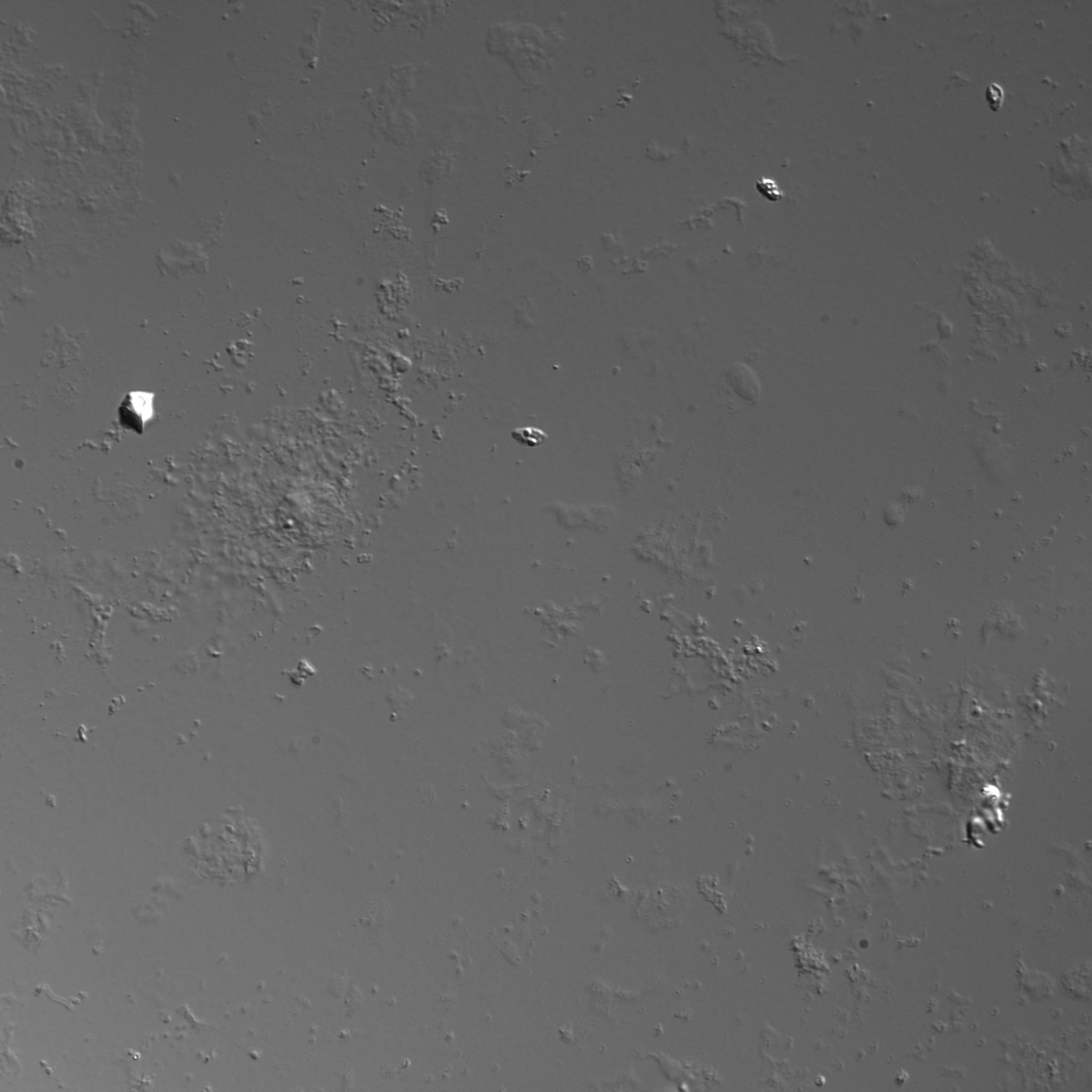

Supplement: Supplementary file 8 — Source Data Fig. 6 [file 44319_2024_84_MOESM8_ESM.zip › 6B/RPTOR1_Cl2_2_DIC_Fig6B.tif]

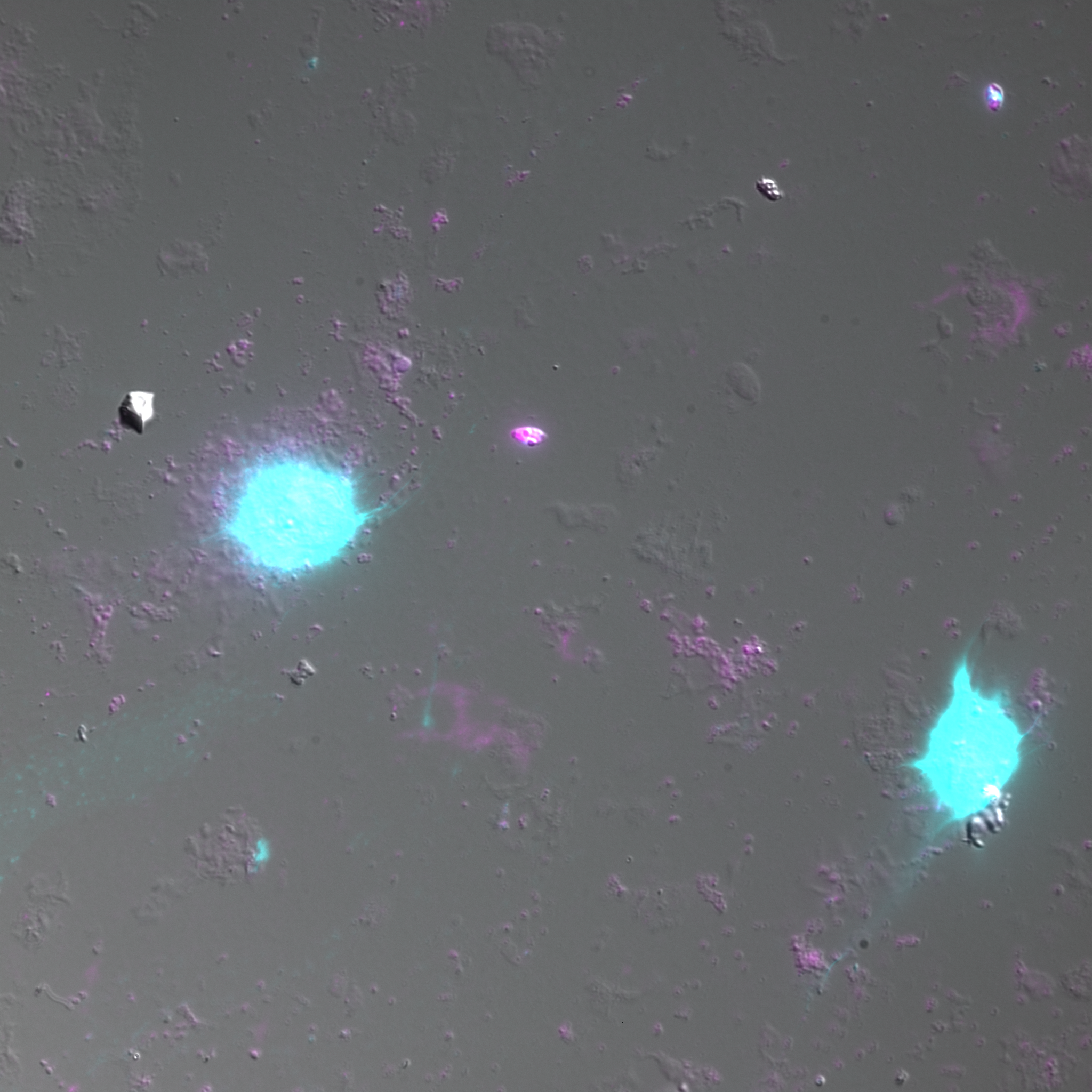

Supplement: Supplementary file 8 — Source Data Fig. 6 [file 44319_2024_84_MOESM8_ESM.zip › 6B/RPTOR1_Cl2_2_Fig6B.tif]

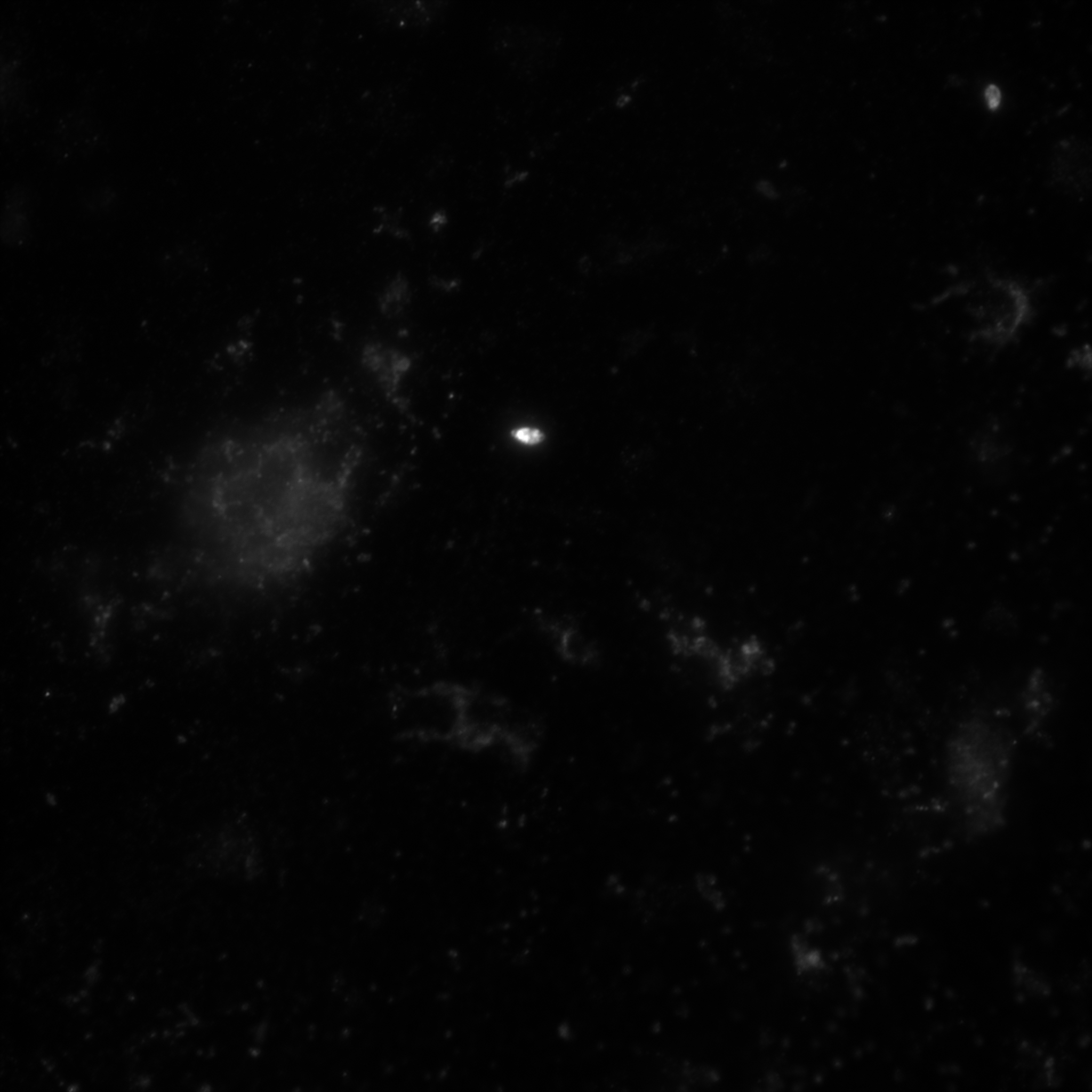

Supplement: Supplementary file 8 — Source Data Fig. 6 [file 44319_2024_84_MOESM8_ESM.zip › 6B/RPTOR1_Cl2_2_OPB_Fig6B.tif]

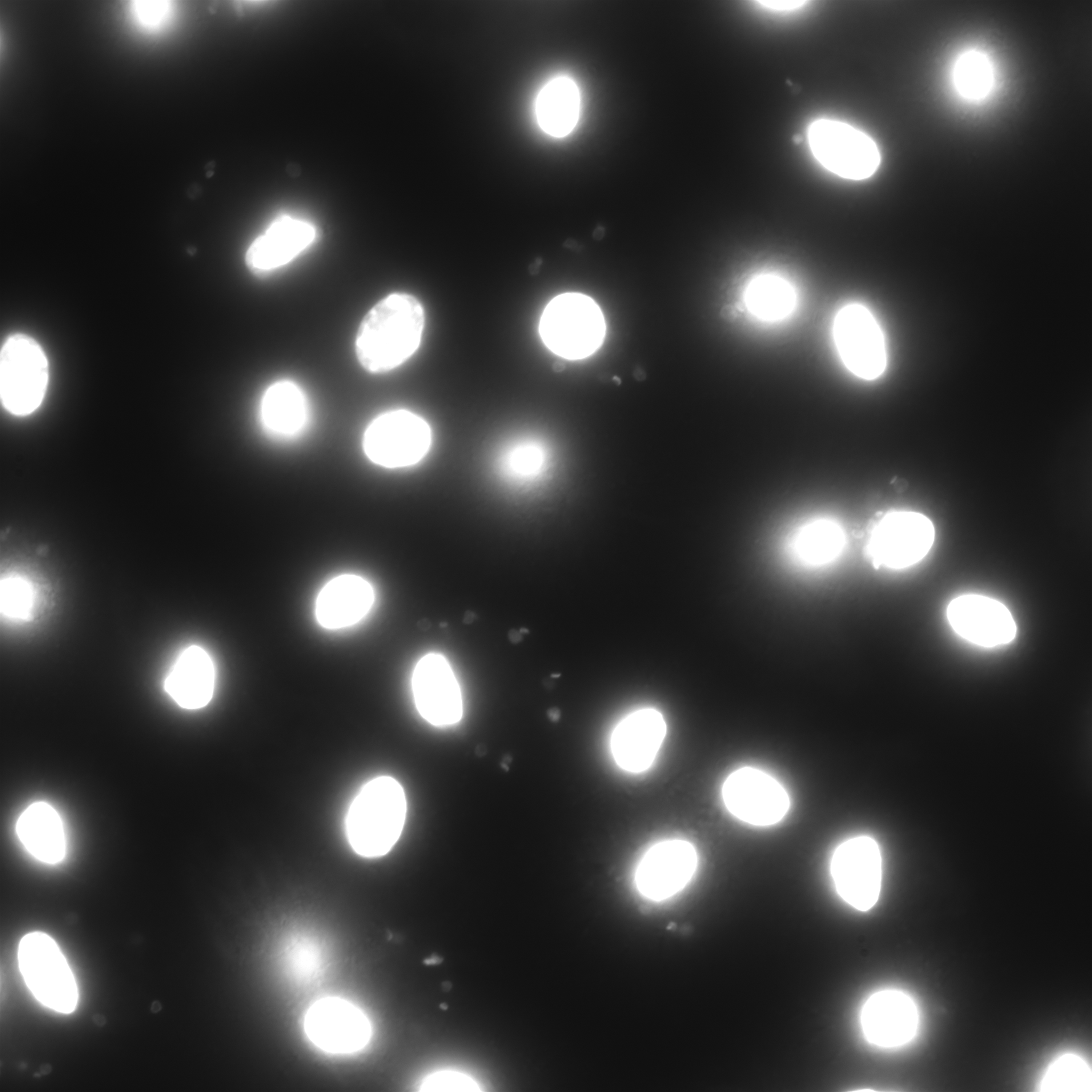

Supplement: Supplementary file 8 — Source Data Fig. 6 [file 44319_2024_84_MOESM8_ESM.zip › 6D/DiCre_ctrl_DAPI.tif]

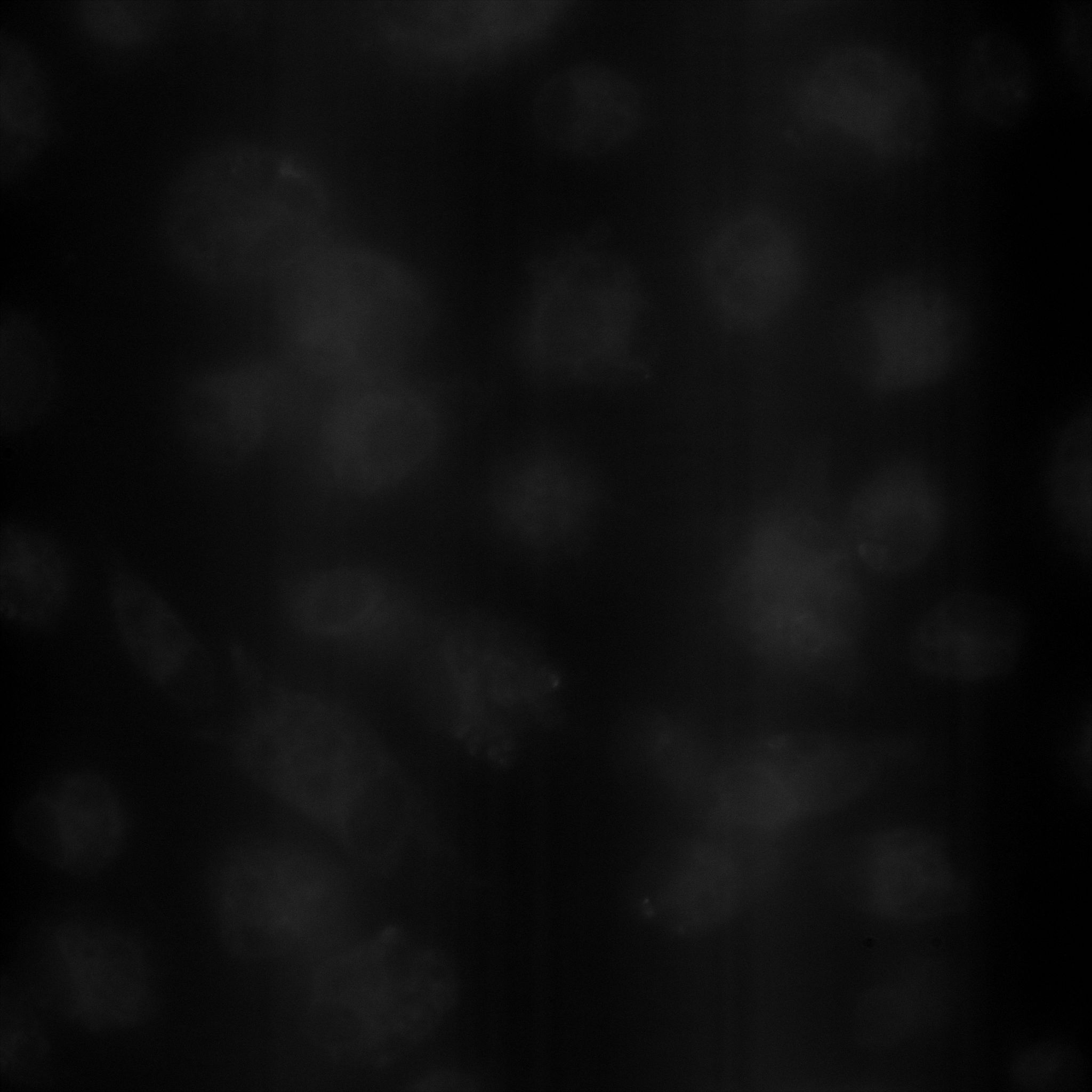

Supplement: Supplementary file 8 — Source Data Fig. 6 [file 44319_2024_84_MOESM8_ESM.zip › 6D/DiCre_ctrl_EdU.tif]

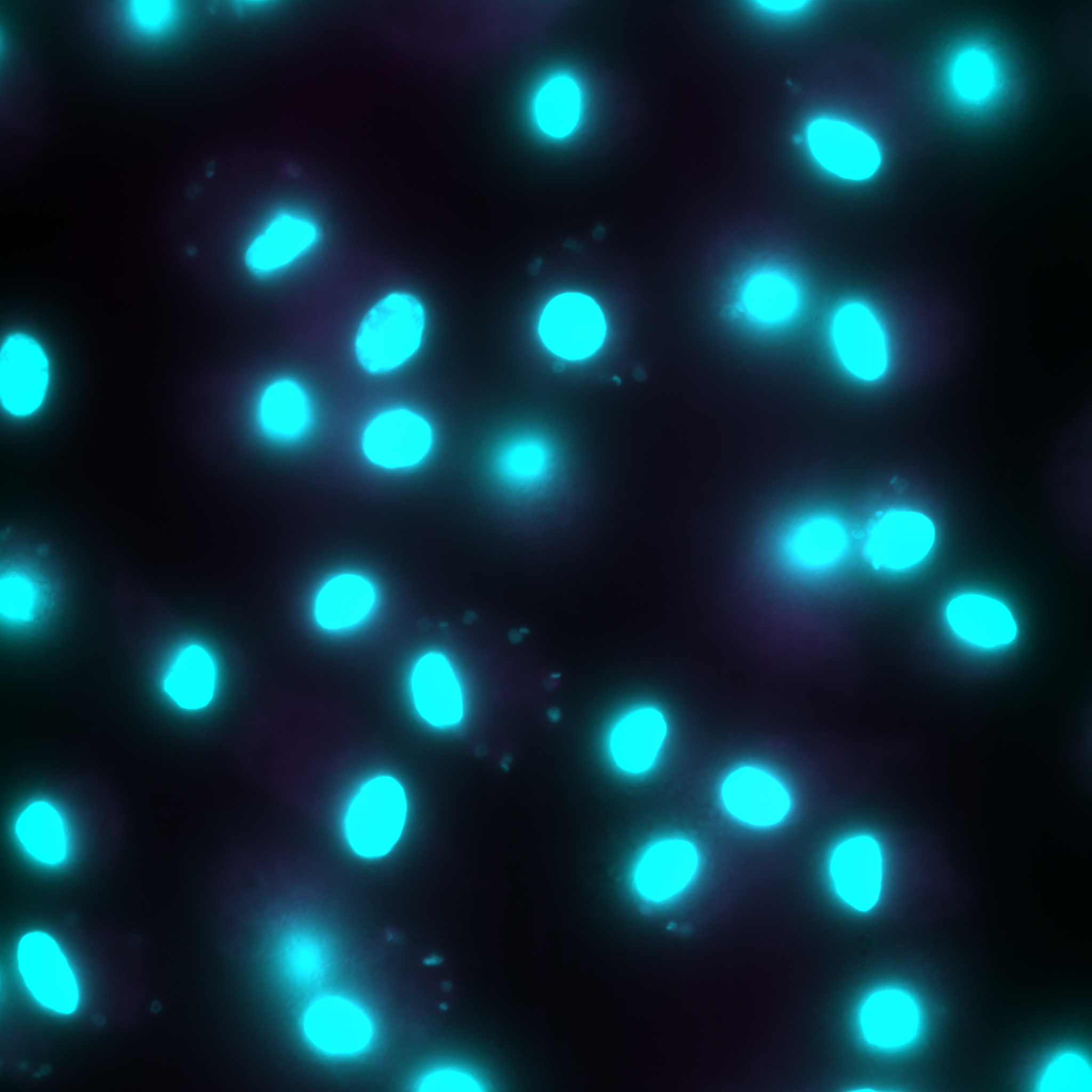

Supplement: Supplementary file 8 — Source Data Fig. 6 [file 44319_2024_84_MOESM8_ESM.zip › 6D/DiCre_ctrl_Merge.tif]

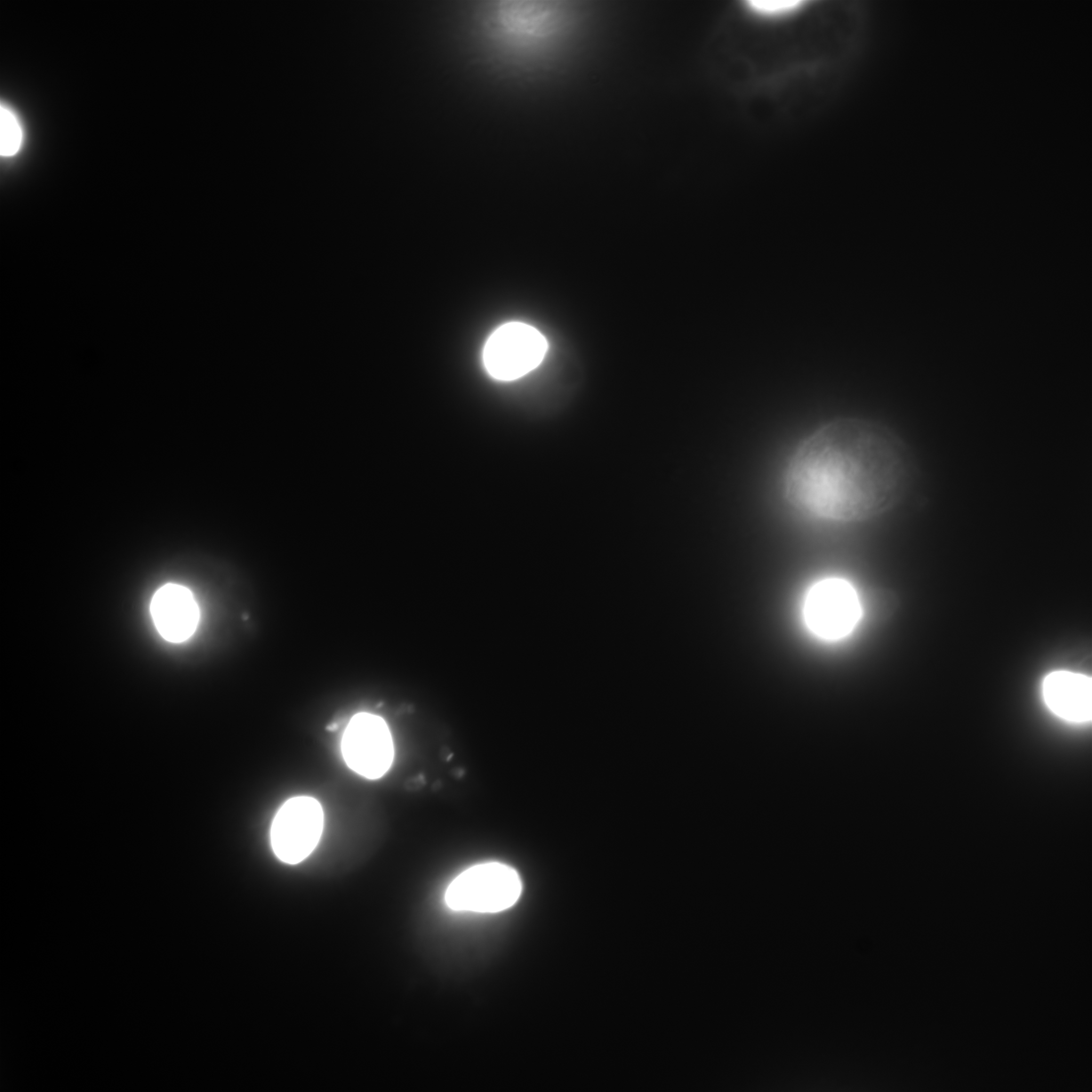

Supplement: Supplementary file 8 — Source Data Fig. 6 [file 44319_2024_84_MOESM8_ESM.zip › 6D/DiCre_EdU_DAPI.tif]

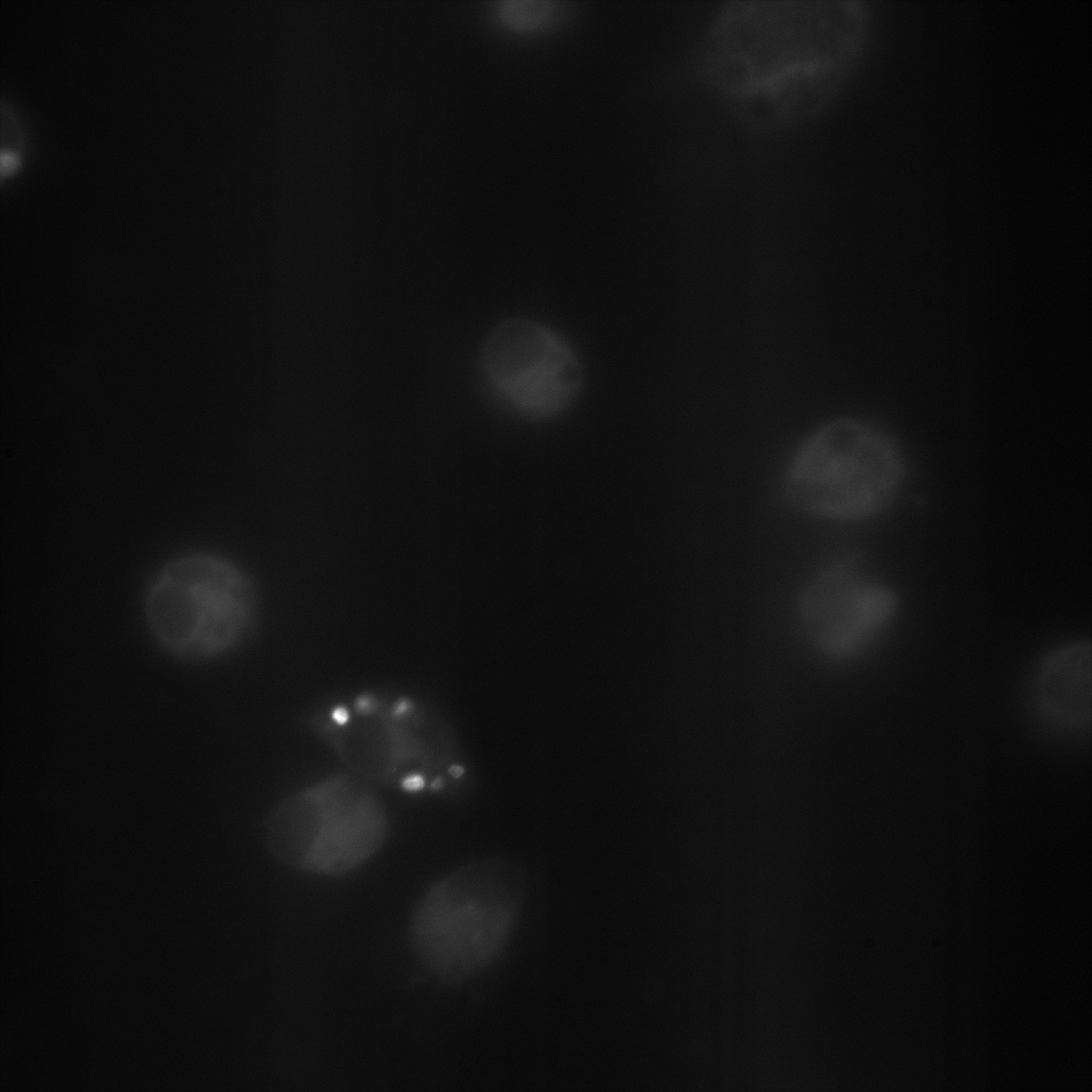

Supplement: Supplementary file 8 — Source Data Fig. 6 [file 44319_2024_84_MOESM8_ESM.zip › 6D/DiCre_EdU_EdU.tif]

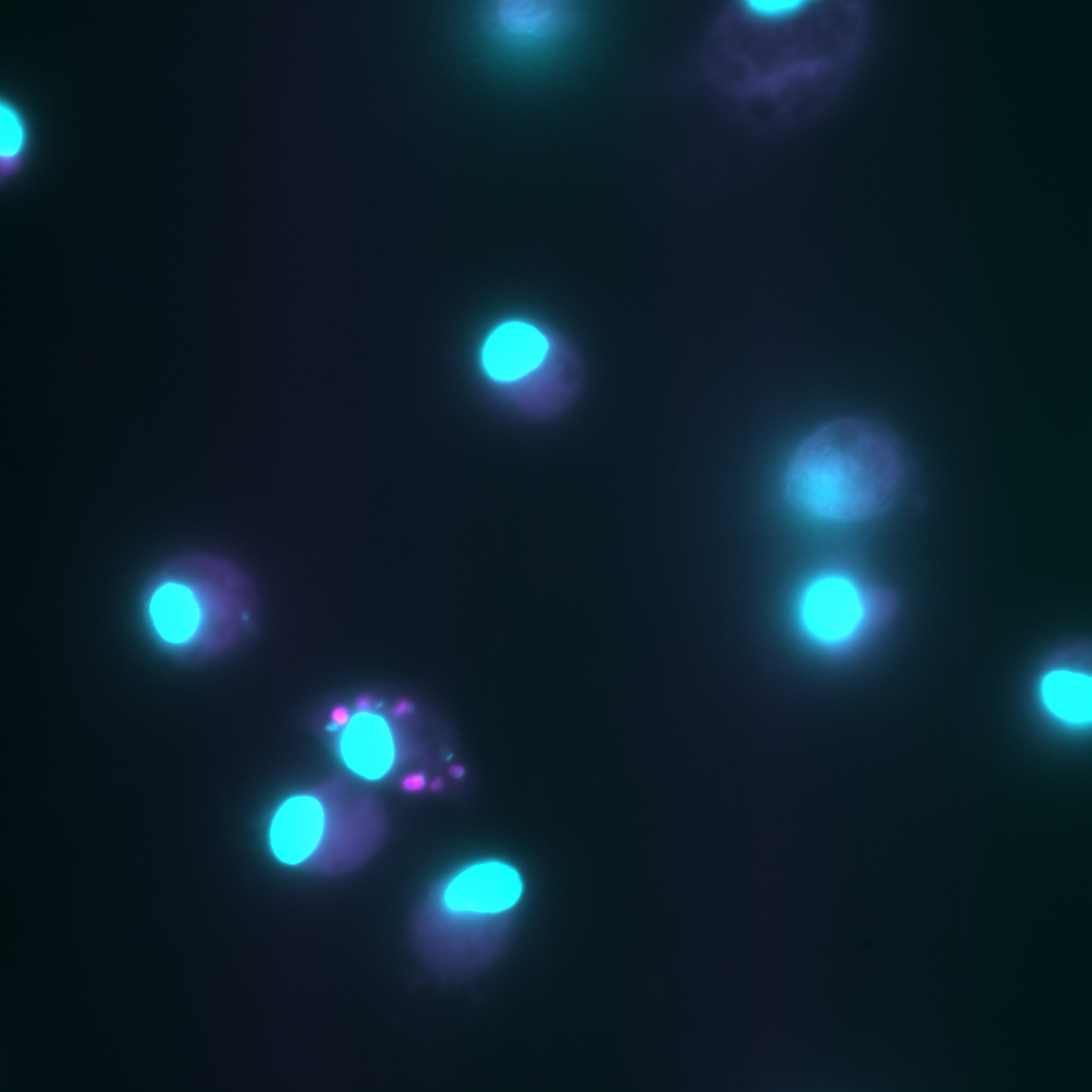

Supplement: Supplementary file 8 — Source Data Fig. 6 [file 44319_2024_84_MOESM8_ESM.zip › 6D/DiCre_EdU_Merge.tif]

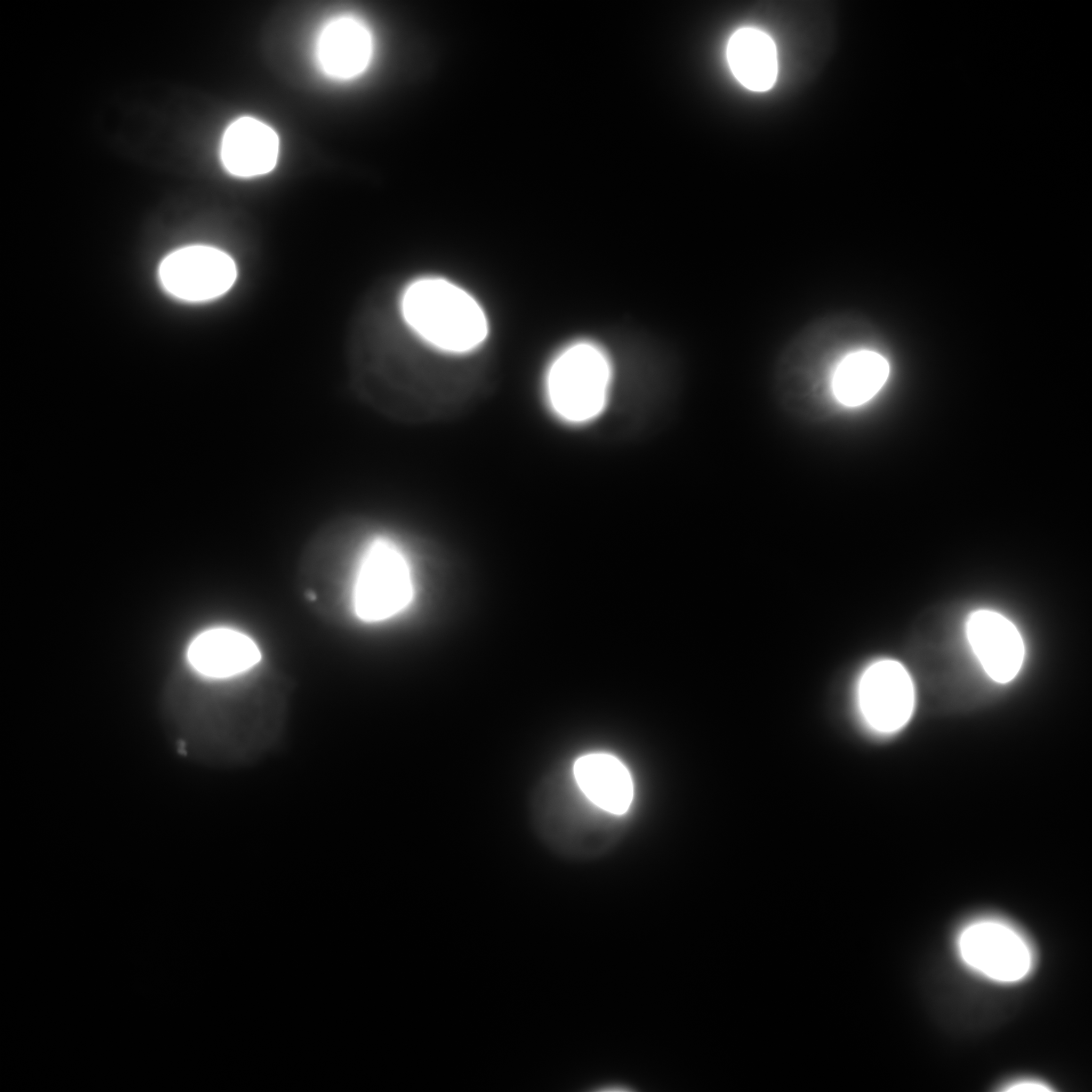

Supplement: Supplementary file 8 — Source Data Fig. 6 [file 44319_2024_84_MOESM8_ESM.zip › 6D/RPTOR1KO_EdU_DAPI.tif]

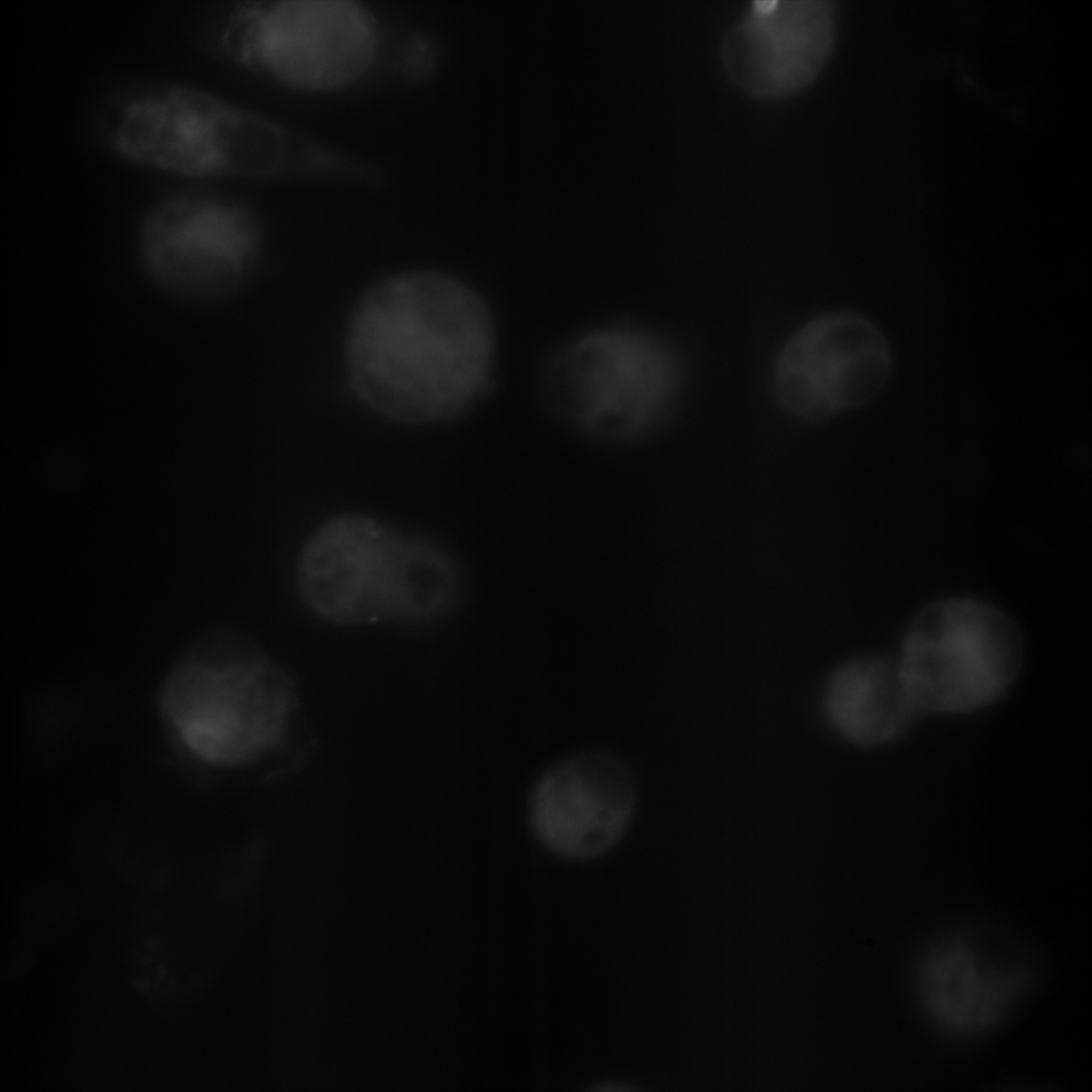

Supplement: Supplementary file 8 — Source Data Fig. 6 [file 44319_2024_84_MOESM8_ESM.zip › 6D/RPTOR1KO_EdU_EdU.tif]

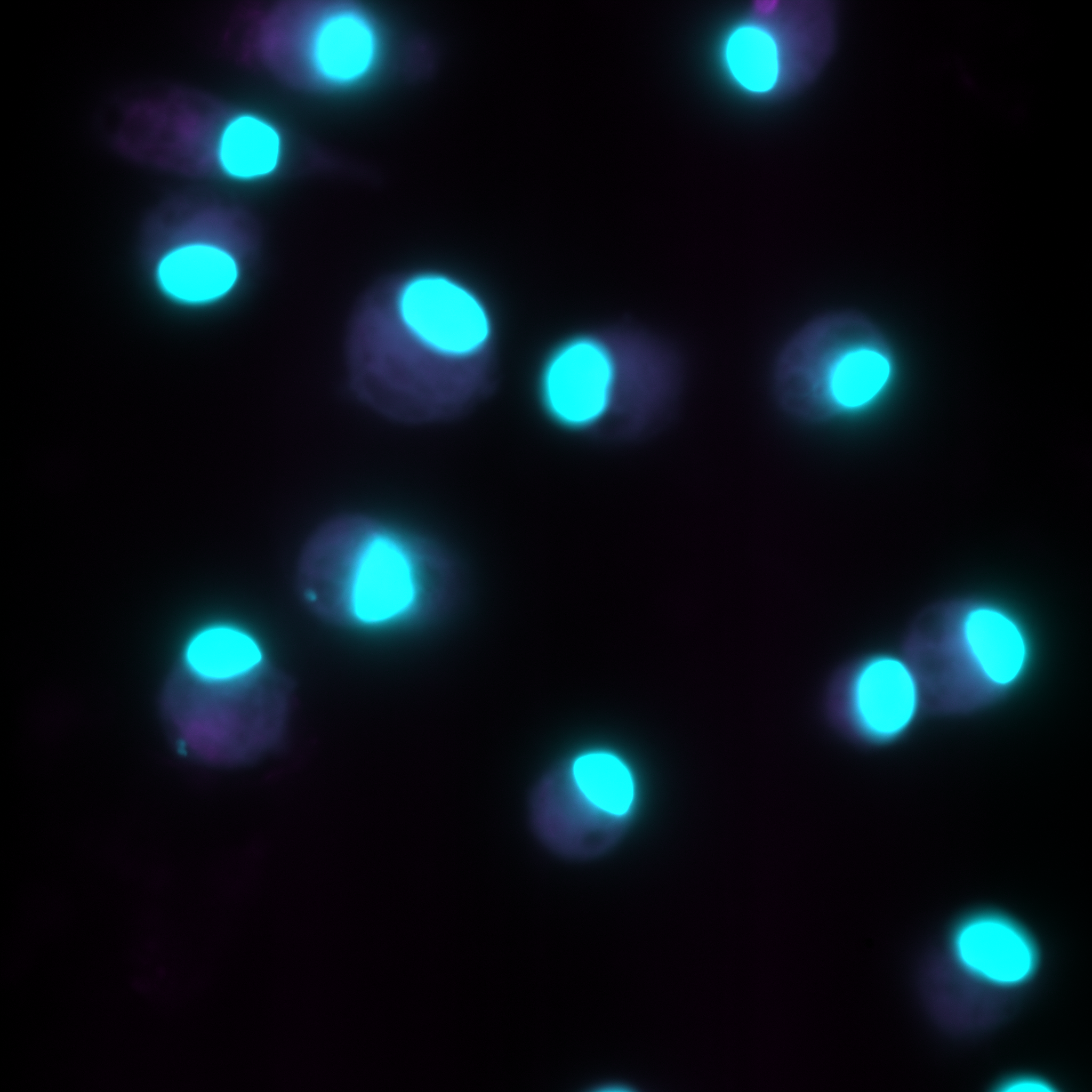

Supplement: Supplementary file 8 — Source Data Fig. 6 [file 44319_2024_84_MOESM8_ESM.zip › 6D/RPTOR1KO_EdU_Merge.tif]

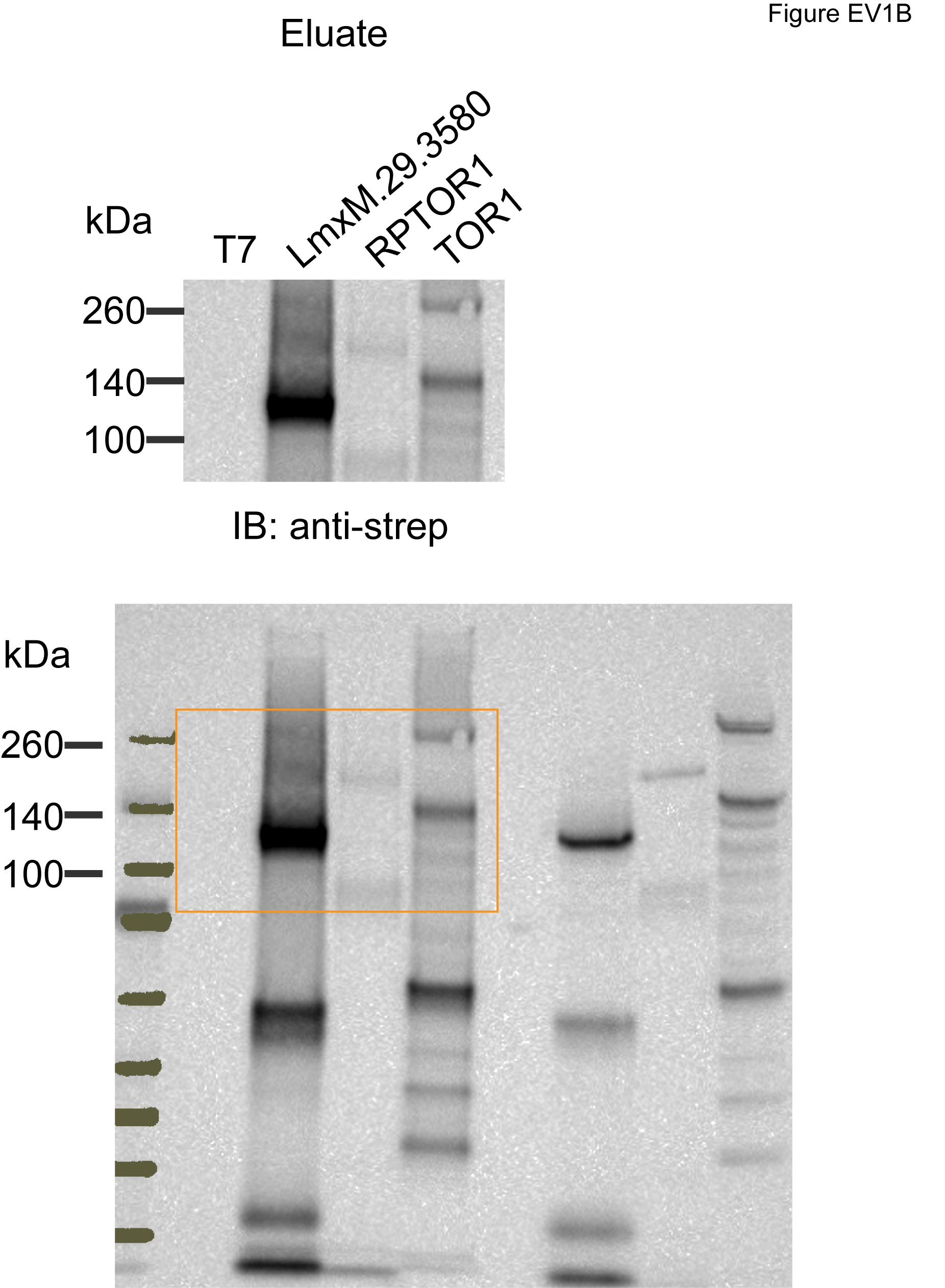

Supplement: Supplementary file 9 — Source data for Expanded View Figures [file 44319_2024_84_MOESM9_ESM.zip › EV1B/EV1B_western_strep_Eluate.tif]

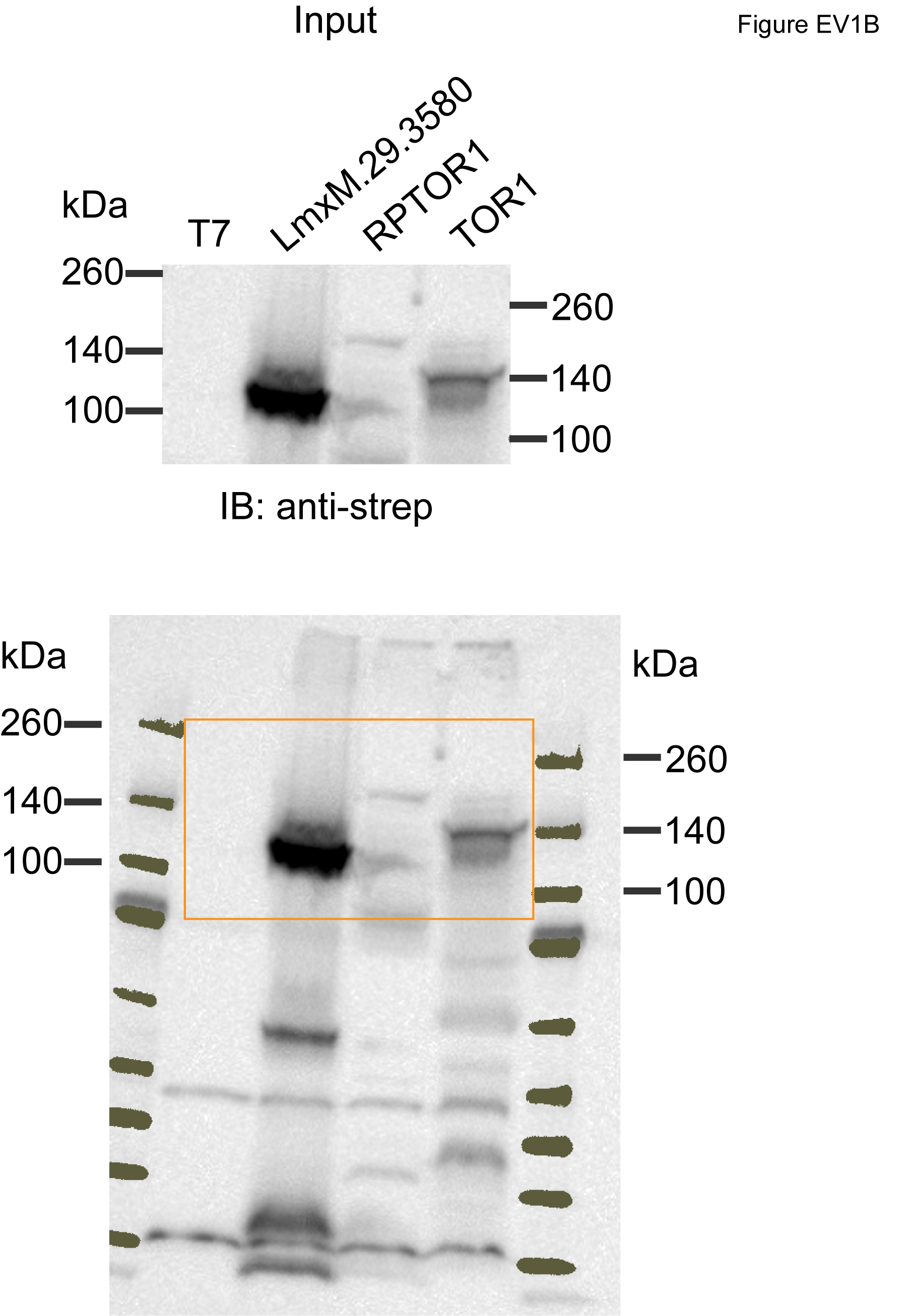

Supplement: Supplementary file 9 — Source data for Expanded View Figures [file 44319_2024_84_MOESM9_ESM.zip › EV1B/EV1B_western_strep_Input.tif]

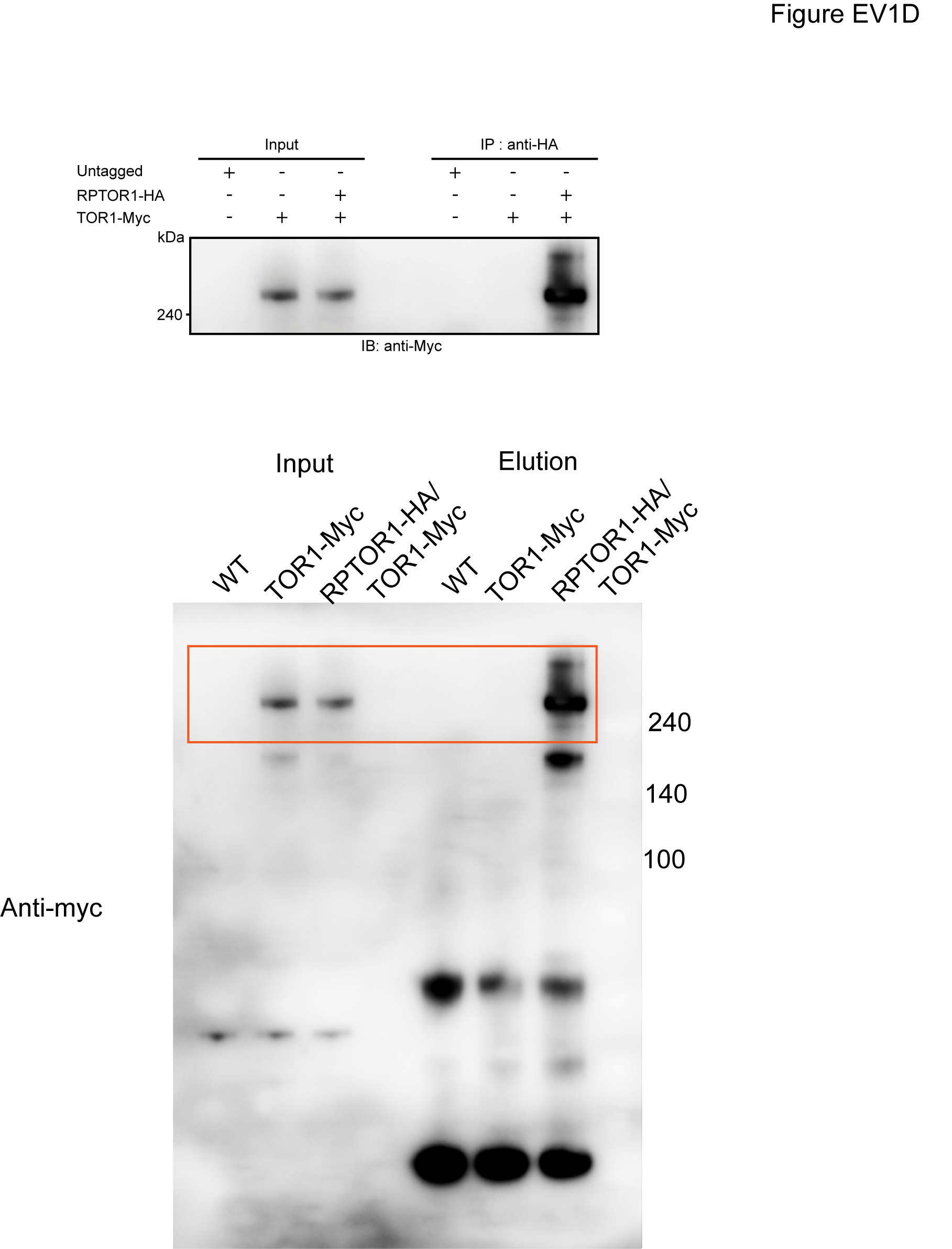

Supplement: Supplementary file 9 — Source data for Expanded View Figures [file 44319_2024_84_MOESM9_ESM.zip › EV1D/EV1D_western_myc.tif]
