# Supplementary material for: TORC1 is an essential regulator of nutrient-controlled proliferation and differentiation in Leishmania
Source: EMBO Rep. 2024 Feb 23;25(3):13. doi: 10.1038/s44319-024-00084-y (PMC10933368; doi:10.1038/s44319-024-00084-y)
Supplement: Supplementary file 11 — Expanded View Figures [file 44319_2024_84_MOESM11_ESM.pdf]

Expanded View Figures

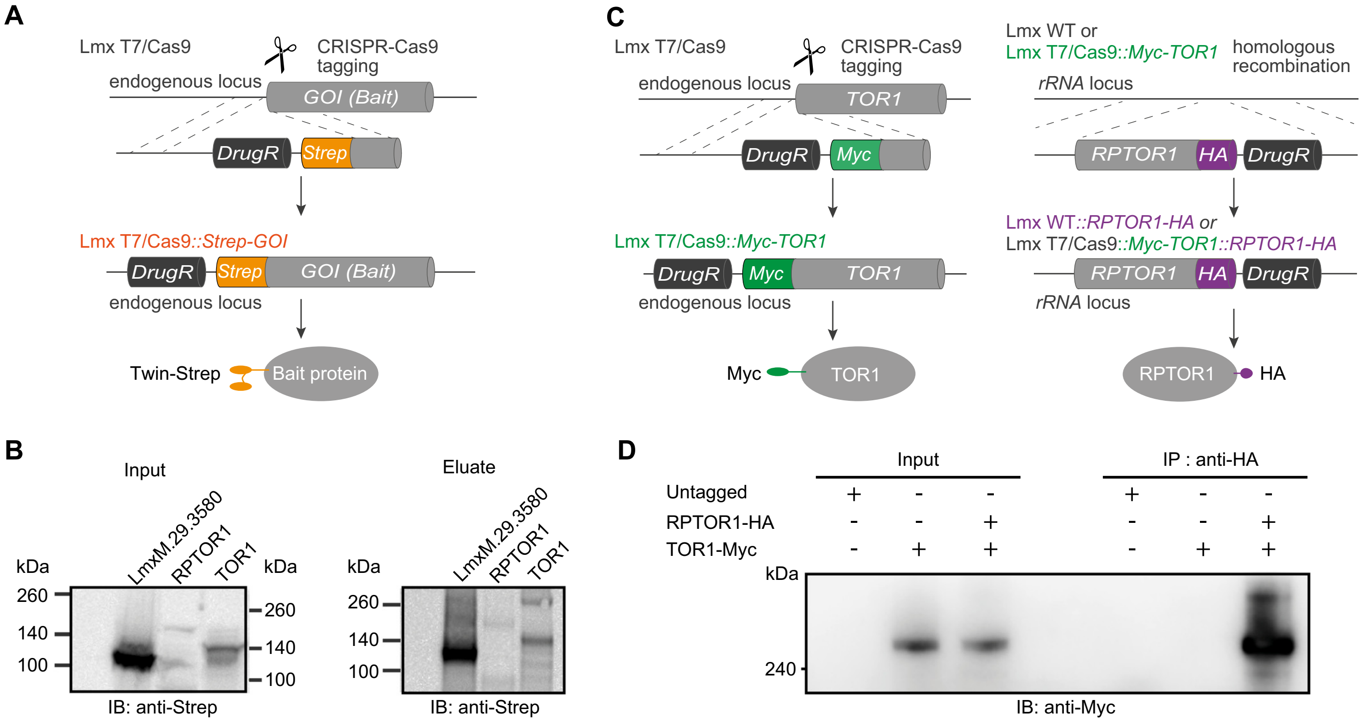

Figure EV1. RPTOR1 immunoprecipitation strategy.

(**A**) Endogenous genes for RPTOR1, TOR1 and control bait LmxM.29.3580 were Twin-Strep-tagged using CRISPR-Cas9 in *L. mexicana* for the affinity purification of these bait proteins and their interactors. GOI: Gene of Interest. (**B**) A sample of lysate, equivalent to 2.25×10^7 cells per line from the *L. mexicana* parental line (T7) or Twin-Strep-tagged lines was taken prior to affinity purification (input) and analysed by western blot. Bait proteins were eluted from MagStrep XT resin with biotin and half of the eluate loaded for analysis by western blot (eluate). Predicted sizes are: LmxM.29.3580 85 kDa, RPTOR1 161 kDa, TOR1 291 kDa. IB: Immunoblot. (**C**) Endogenous *TOR1* was Myc-tagged using a CRISPR-Cas9 tagging approach while HA-tagged *RPTOR1* was inserted in the ribosomal locus using homologous recombination to generate single or dual-tagged *L. mexicana* lines. (**D**) Lysates of *L. mexicana* expressing untagged RPTOR1 and TOR1, HA-tagged RPTOR1 and/or Myc-tagged TOR1 were incubated with anti-HA-conjugated magnetic beads. Input samples were removed before addition of beads and 20 µg of protein loaded for each sample. After 6 washes, beads were eluted in 50 µL laemmli buffer per line and half of this eluate analysed by western blot using anti-Myc antibodies. IB: Immunoblot. Source data are available online for this figure.

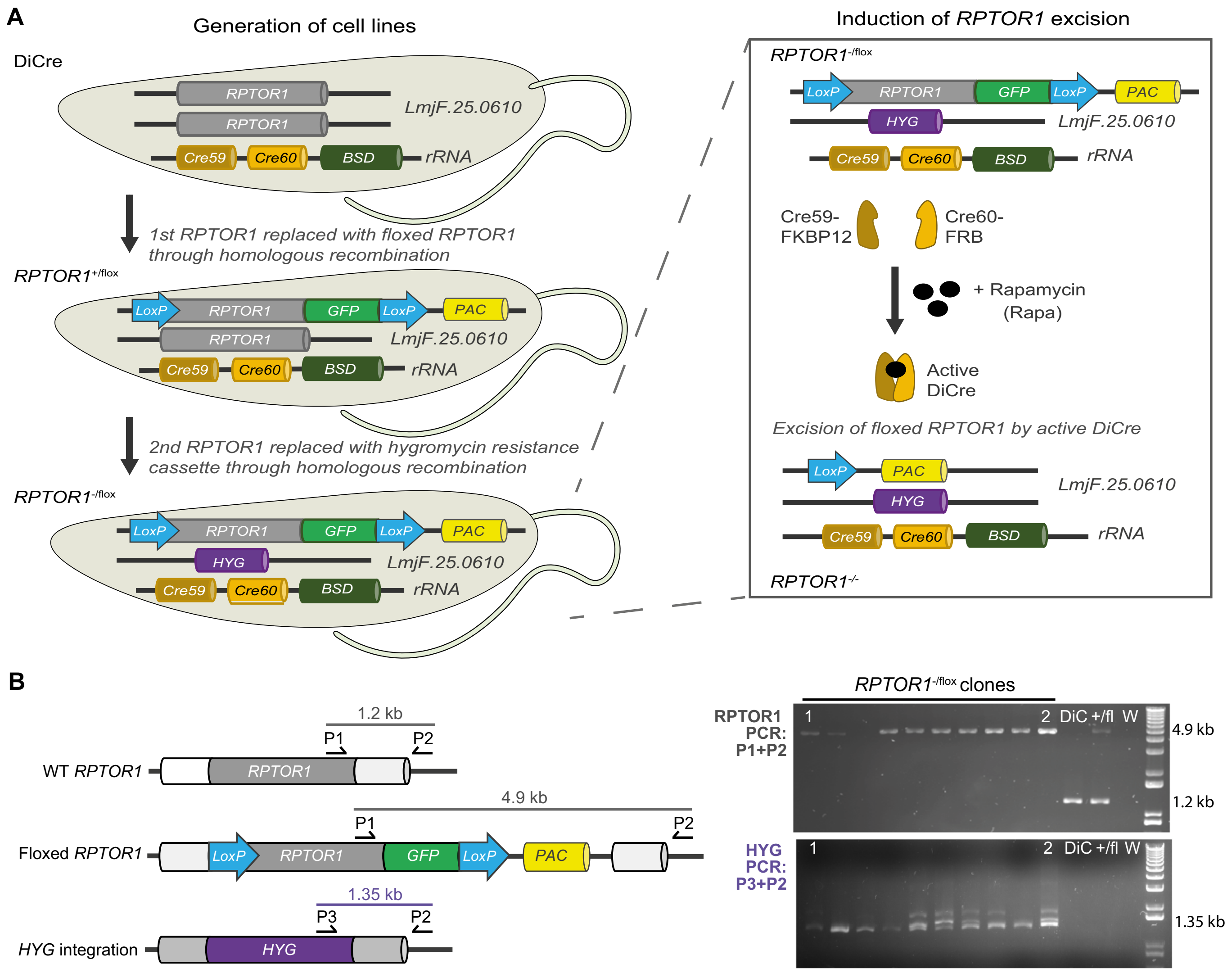

Figure EV2. RPTOR1 knockout strategy.

(**A**) Schematic of *RPTOR1* knockout strategy. The background cell line, DiCre, was generated by integrating the diCre expression cassette into the ribosomal RNA locus for constitutive expression of FKBP-Cre59 and FRB-Cre60 in *L. major* Friedlin. The inducible *RPTOR1* knockout line (*RPTOR1*$^{-/flox}$) was generated by replacing the 1st *RPTOR1* allele with a LoxP flanked (floxed) C-terminal GFP-tagged version of *RPTOR1* followed by replacement of the 2nd allele with a hygromycin resistance cassette. The floxed *RPTOR1* gene can be excised by Cre-recombinase following rapamycin induced dimerization to generate a *RPTOR1*$^{-/-}$ line. (**B**) Diagnostic PCRs of gDNA from generated cell lines confirm integration of hygromycin resistance and floxed *RPTOR1* cassettes. Primer binding site and size of PCR products are shown in the diagram (left). Ten clones of *RPTOR1*$^{-/flox}$ are shown after PCR and agarose gel electrophogresis (right); lanes with clones 1 and 2 that are described in this study are indicated on the gel image. DiC, DiCre; +/fl, *RPTOR1*$^{+/flox}$; W, water control.

© The Author(s)

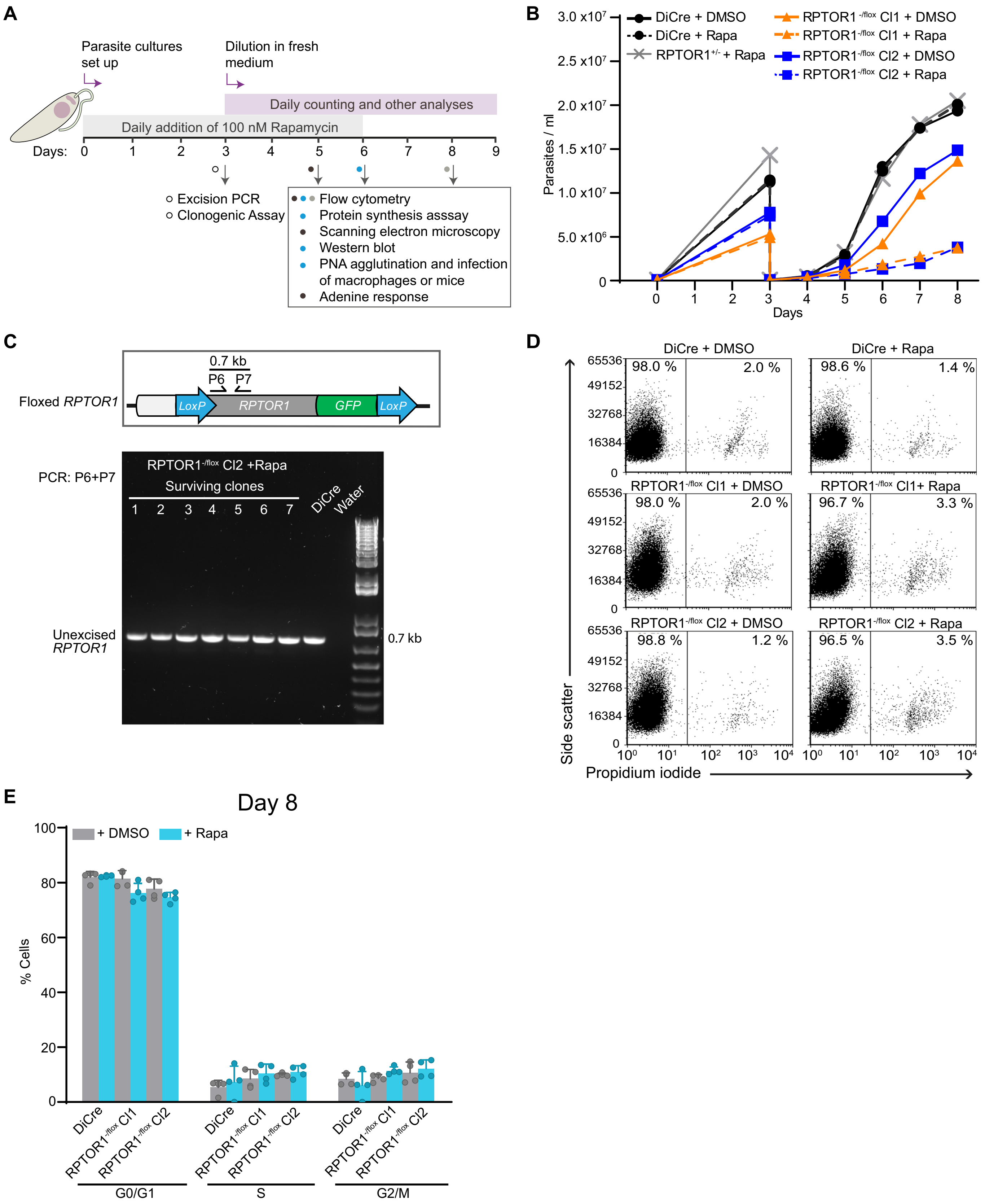
A
Parasite cultures set up
Dilution in fresh medium
Daily counting and other analyses
Daily addition of 100 nM Rapamycin
Days: 0 1 2 3 4 5 6 7 8 9
Excision PCR
Clonogenic Assay
Flow cytometry
Protein synthesis asssay
Scanning electron microscopy
Western blot
PNA agglutination and infection of macrophages or mice
Adenine response
B
DiCre + DMSO
DiCre + Rapa
RPTOR1+/- + Rapa
RPTOR1-/flox Cl1 + DMSO
RPTOR1-/flox Cl1 + Rapa
RPTOR1-/flox Cl2 + DMSO
RPTOR1-/flox Cl2 + Rapa
Parasites / ml
Days
C
Floxed RPTOR1
0.7 kb
P6 P7
LoxP RPTOR1 GFP LoxP
PCR: P6+P7
RPTOR1-/flox Cl2 +Rapa
Surviving clones
1 2 3 4 5 6 7 DiCre Water
Unexcised RPTOR1
0.7 kb
D
DiCre + DMSO
DiCre + Rapa
RPTOR1-/flox Cl1 + DMSO
RPTOR1-/flox Cl1+ Rapa
RPTOR1-/flox Cl2 + DMSO
RPTOR1-/flox Cl2 + Rapa
98.0 % 2.0 %
98.6 % 1.4 %
98.0 % 2.0 %
96.7 % 3.3 %
98.8 % 1.2 %
96.5 % 3.5 %
Side scatter
Propidium iodide
E
Day 8
+ DMSO + Rapa
% Cells
DiCre
RPTOR1-/flox Cl1
RPTOR1-/flox Cl2
G0/G1
S
G2/M

Figure EV3. RPTOR1 is essential for cell proliferation and long-term survival.

(**A**) Schematic of the rapamycin induction and analysis timeline. Log-stage promastigotes were set up at 1×10^5 cells mL^{-1} (day 0) and treated for three days with daily addition of DMSO or 100 nM rapamycin; cells were then counted and diluted to 1×10^5 cells mL^{-1} (day 3) followed by culturing for up to 6 additional days for daily counting and other analyses as indicated. (**B**) Cell densities of uninduced (+DMSO, solid line) and rapamycin-induced (+Rapa, dashed line) cells. Promastigotes of DiCre (black), *RPTOR1*$^{+/-}$ (grey, +Rapa only) and *RPTOR1*$^{-/flox}$ lines, Cl1 (orange) and Cl2 (blue) were counted daily for five days after the initial three days of rapamycin induction. A representative dataset of three to four similar experiments is shown. (**C**) PCR analysis of genomic DNA from surviving clones (clones 1–7) of rapamycin-induced *RPTOR1*$^{-/flox}$ Cl2 and DiCre cells. Schematic (upper panel) shows the *RPTOR1* locus with floxed *RPTOR1* cassette with primer binding sites and the predicted length of the PCR amplicon. Agarose gel (lower panel) indicates the presence of the *RPTOR1* CDS fragment from the unexcised floxed *RPTOR1* cassette in the seven clones from two clonogenic assays. (**D**) Cell viability was measured by flow cytometry of propidium iodide-stained cells after five days of induction. Representative dot plots of side scatter versus propidium iodide fluorescence are shown for uninduced (+DMSO) or rapamycin-induced (+Rapa) cells. Numbers indicate the percentages of cells within the gate with live cells shown in the propidium iodide negative (left) gate in each plot. (**E**) Cell cycle analysis of fixed propidium iodide cells eight days after induction. Data show mean ± SD ($n = 4$ biological replicates) from two experiments; each dot denotes a replicate. Source data are available online for this figure.

 © The Author(s)

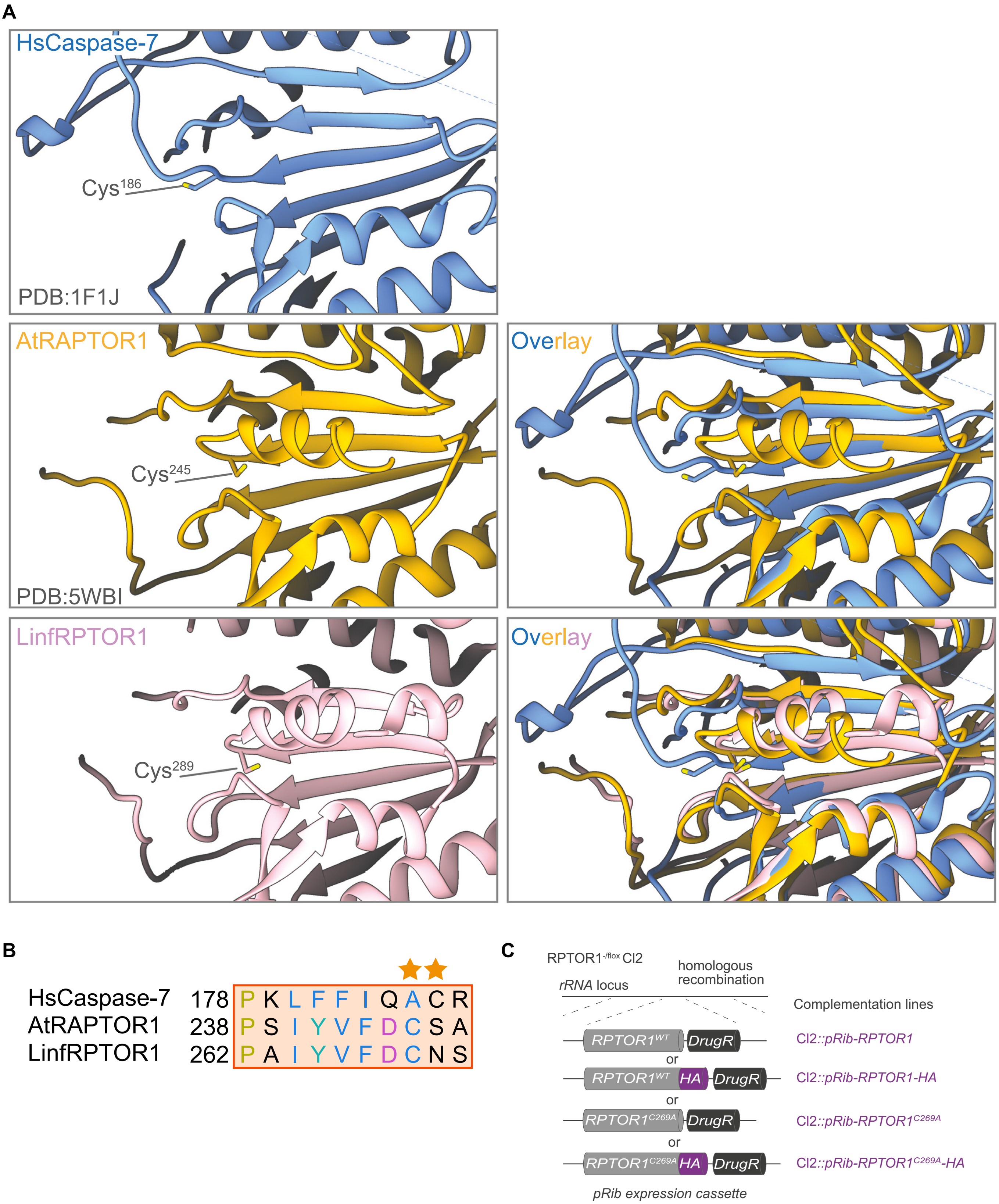
A
HsCaspase-7
Cys186
PDB:1F1J
AtRAPTOR1
Cys245
PDB:5WBI
Overlay
LinfRPTOR1
Cys289
Overlay
B
HsCaspase-7 178 P K L F F I Q A C R
AtRAPTOR1 238 P S I Y V F D C S A
LinfRPTOR1 262 P A I Y V F D C N S
C
RPTOR1-/flox Cl2
rRNA locus
homologous recombination
Complementation lines
RPTOR1WT DrugR
Cl2::pRib-RPTOR1
or
RPTOR1WT HA DrugR
Cl2::pRib-RPTOR1-HA
or
RPTOR1C269A DrugR
Cl2::pRib-RPTOR1C269A
or
RPTOR1C269A HA DrugR
Cl2::pRib-RPTOR1C269A-HA
pRib expression cassette

Figure EV4. Secondary sequences alignments using Alphafold model of RPTOR1.

(**A**) The X-ray crystal structures of HsCaspase-7 (PDB:1F1J), AtRAPTOR1 (PDB:5WBI), and the Alphafold model of LinfRPTOR1 (LINF_250011400) are shown individually on the left hand panels. The active site cysteine residues are denoted by the labels. The AlphaFold model (AF-A4I1A2-F1-model_v3.pdb) was downloaded from the AlphaFold Protein Structure Database (AlphafoldDB). AtRAPTOR1 and LinfRPTOR1 were superposed in UCSF Chimera using the MatchMaker tool (right hand panels). (**B**) The Match->Align tool was used to generate amino acid alignments from the structural superposition. Residues are coloured using the ClustalX scheme. The active site cysteines are annotated by stars above the sequence. (**C**) RPTOR1 complementation lines were generated by integrating untagged or HA-tagged WT and C269A *RPTOR1* into the ribosomal locus of *RPTOR1*$^{-/flox}$ Cl2 using homologous recombination.

© The Author(s)

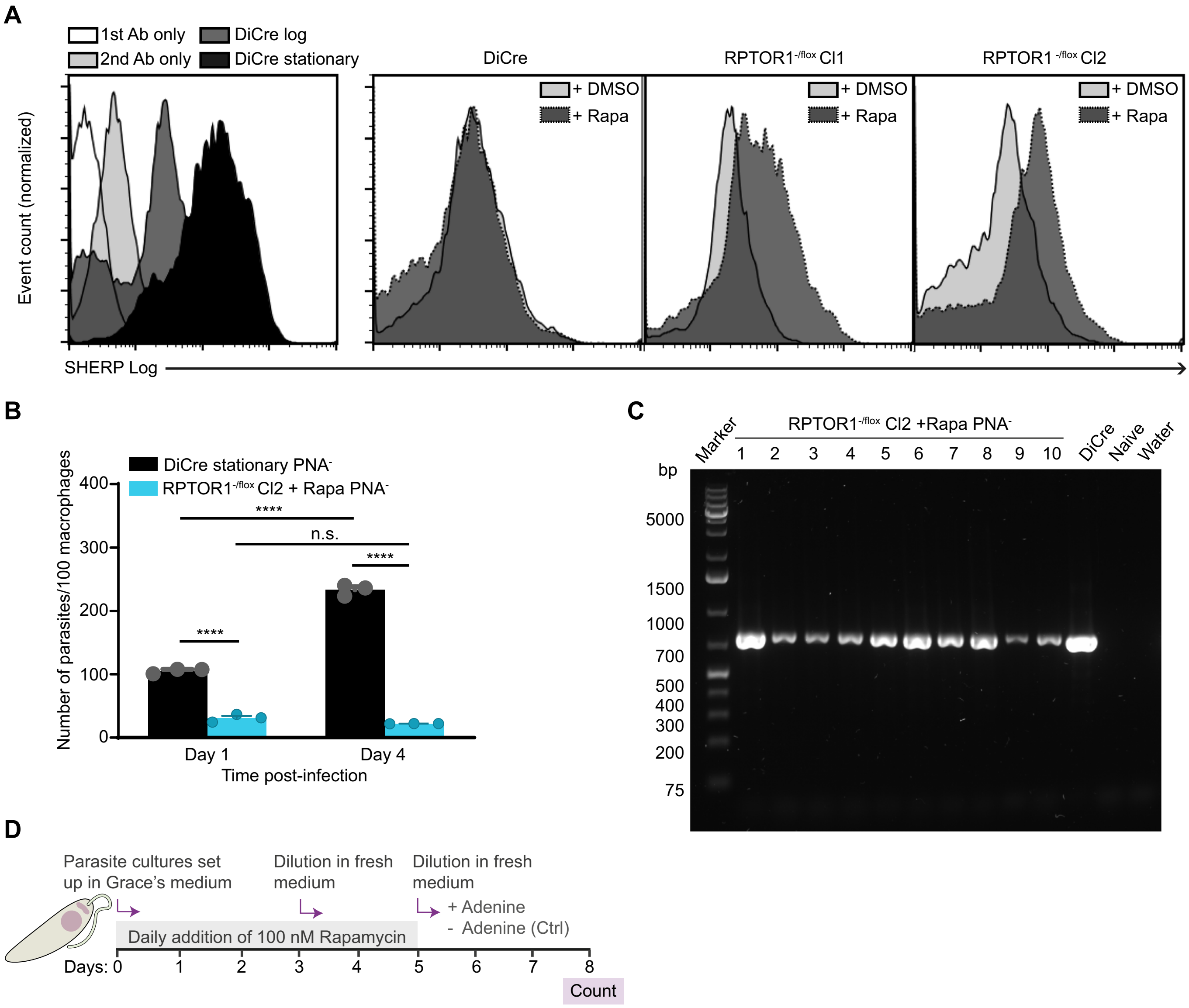

Figure EV5. RPTOR1 loss induces metacyclogenesis but is detrimental for murine infection.

(**A**) Flow cytometry analysis of SHERP expression. Log-stage promastigotes were treated for three days with DMSO or rapamycin (+Rapa), diluted and cultured for three more days with daily DMSO and rapamycin treatment. Cells were then fixed, permeabilized and stained with anti-SHERP and Alexa Fluor 647 (AF647)-conjugated secondary antibodies. Histograms of AF647 fluorescence (SHERP staining) in control (left panel) and DMSO or rapamycin treated cells. Controls (left panel) include DiCre cells stained with primary (1st Ab) or secondary antibody (2nd Ab) only and SHERP-stained DiCre early-log or stationary-phase cells. (**B**) Macrophage infectivity of PNA$^-$ promastigotes. DiCre cells were cultured for 7 days in the presence of rapamycin to reach stationary phase. *RPTOR1$^{-/flox}$* cells were induced with rapamycin for three days, diluted in fresh medium and cultured for three more days with daily addition of rapamycin. Metacyclic promastigotes (PNA$^-$) were then purified from cultures by PNA agglutination and added to thioglycollate-elicited peritoneal macrophages at a 6:1 ratio (parasites:macrophages). Cells were analysed at day 1 and day 4 after infection using microscopy. Graph shows values of triplicate wells from one experiment as dots and their mean ± SD. A repeat experiment using an infection ratio of 3:1 is shown in Fig. 6A. ****P* value ≤ 0.001 in a two-way ANOVA with Bonferroni post hoc test. n.s. not significant. (**C**) PCR analysis of genomic DNA from ears of BALB/c mice that were infected with PNA$^-$ promastigotes. Ears were harvested at 6 wks p.i., digested with collagenase D and the genomic DNA extracted. To assess the presence of escape mutants (with un-excised RPTOR1) PCR was performed using primers that amplify a 700 bp fragment from the RPTOR1 CDS. gDNA from the ears of ten mice infected with rapamycin-induced *RPTOR1$^{-/flox}$*, one mouse infected with DiCre (positive control), one naïve mouse (negative control) or water (negative control) was used as template. Samples originated from the same ears represented in Fig. 6F and Fig. 6G. (**D**) Outline of adenine response experiment. DiCre, *RPTOR1$^{-/flox}$* Cl2 and *RPTOR1* complementation lines were grown in Grace's medium with daily addition of 100 nM rapamycin or DMSO for five days. After three days of induction cells were diluted in fresh medium. On day 5 cells were diluted again with the addition or not of 500 μM adenine and counted after three days (day 8). Source data are available online for this figure.
